# Patient-reported symptom profiles in vitamin B_12_ deficiency and pernicious anemia: a cross-sectional study using latent class analysis

**DOI:** 10.3389/fmed.2026.1927721

**Published:** 2026-09-18

**Authors:** Brooke M. Morris, Alfie Thain, Blair Coe Schweiger, Ebba Nexø, Andrew McCaddon, Bruce H. R. Wolffenbuttel, Ralph Green, David H. Alpers, Marie-Joe Dib, Petra Visser, Katrina Burchell, Kourosh R. Ahmadi, Austin W. Reynolds

**Affiliations:** 1 Department of Biology, Baylor University, Waco, TX, United States; 2 School of Biosciences and Medicine, University of Surrey, Guildford, United Kingdom; 3 Department of Anthropology, Baylor University, Waco, TX, United States; 4 Department of Clinical Biochemistry, Aarhus University Hospital, Aarhus, Denmark; 5 Faculty of Social and Life Sciences, Wrexham University, Wrexham, United Kingdom; 6 Department of Endocrinology, University of Groningen, University Medical Center Groningen, Groningen, Netherlands; 7 Department of Pathology and Laboratory Medicine, School of Medicine, University of California, Davis, Davis, CA, United States; 8 Department of Internal Medicine Hematology and Oncology, School of Medicine, University of California, Davis, Davis, CA, United States; 9 Department of Internal Medicine, Division of Gastroenterology, Washington University School of Medicine, St Louis, MO, United States; 10 Division of Cardiovascular Medicine, Perelman School of Medicine, University of Pennsylvania, Philadelphia, PA, United States; 11 The Pernicious Anaemia Society, Ipswich, United Kingdom; 12 Department of Microbiology, Immunology, and Genetics, University of North Texas Health Science Center at Fort Worth, Fort Worth, TX, United States

**Keywords:** latent class analysis, pernicious anemia, symptom burden, treatment satisfaction, vitamin B_12_ deficiency

## Abstract

Pernicious anemia is a chronic autoimmune disease characterized by impaired of vitamin B_12_ absorption and deficiency. Current diagnostic approaches rely on laboratory biomarkers with limitations in sensitivity and specificity, leading to diagnostic uncertainty and potentially delayed and/or suboptimal treatment. The 2024 NICE guidelines acknowledge that treatment frequency should be guided by individual symptom response rather than current one-size-fits-all schedules; however, the symptom heterogeneity underpinning this recommendation remains poorly characterized. We conducted secondary analysis of symptom survey data from 1,117 members of the Pernicious Anaemia Society (PAS) collected between August 2010 and November 2012. We applied latent class analysis (LCA) to 46 self-reported indicators comprising demographic characteristics, symptoms, and comorbid conditions, to identify distinct symptom-based subtypes and assess whether such subgroups can inform more tailored management of PA. Associations between symptom subtypes and diagnostic test results, age when symptoms first started, symptom duration, and treatment satisfaction were examined using chi-square and Fisher’s exact tests. The best model included three distinct symptom subtypes: High Burden (*n* = 334, 29.9%), Moderate Burden (*n* = 613, 54.9%), and Low Burden (*n* = 170, 15.2%). The High Burden subtype exhibited fatigue (99.4%), cognitive dysfunction (97.9% memory loss), neurological manifestations (95.5% clumsiness), and suicidal thoughts (41.6%). Intrinsic factor antibody (IFA) status did not differ significantly across subtypes (*χ*^2^ = 1.04, df = 2, *p* = 0.593). Age at symptom onset differed significantly across subtypes (*p* = 0.002), with the Low Burden subtype overrepresented in older age groups. The High Burden subtype had the longest diagnostic delays (56.9% >2 years). Standard three-monthly injections showed low satisfaction across all classes (High Burden: 8.5%, Moderate Burden: 23.7%, Low Burden: 30.7%). Symptom-based stratification identifies clinically meaningful subgroups independent of IFA status, supporting tailored, symptom-guided treatment rather than the current piecemeal approach. The inverse association between high symptom burden and age of onset warrants clinical attention. The co-occurrence of markedly elevated rates of depression and suicidal thoughts in the High Burden subtype suggest that integrated mental health assessments should form part of routine clinical management.

## Introduction

Vitamin B_12_ is an essential micronutrient obtained almost exclusively from animal products. It plays critical roles in numerous physiological processes including myelination, DNA synthesis, methyltransferase activity and gene expression regulation (1, 2). Deficiency is surprisingly common, affecting approximately 6% of people under age 60 and 10–20% of the elderly population (3, 4). B_12_ deficiency can result from various factors including malabsorption disorders, medication effects (proton-pump inhibitors and metformin), and vegetarian or vegan diets (5, 6). A particularly concerning cause is autoimmune destruction of an essential component for the normal B_12_ absorption pathway, leading to pernicious anemia (PA) (7).

PA represents the end-stage of autoimmune gastritis (AIG), a complex and heterogeneous condition in which antibodies target gastric parietal cells in the stomach fundus and corpus (8–10). These cells produce intrinsic factor, a protein essential for B_12_ absorption in the small intestine (11). Without treatment, the resulting B_12_ deficiency progressively leads to neurological dysfunction, cognitive decline, severely compromised quality of life (QoL), and megaloblastic anemia (12).

Although PA is commonly considered rare, affecting only 0.1% of the general population and 2–3% of older adults, diagnostic challenges lead to significant underestimation of its true prevalence (13, 14). Distinguishing PA from other causes of B_12_ deficiency presents a fundamental clinical challenge, as low B_12_, high methylmalonic acid (MMA) or homocysteine cannot differentiate autoimmune-mediated PA from dietary insufficiency, malabsorption due to medications, or other gastrointestinal conditions. Current recommendations for diagnostic approaches distinguishing PA from other causes of B_12_ deficiency rely on measures of intrinsic factor antibodies (IFA) sometimes including parietal cell antibodies (PCA) and occasionally endoscopy with biopsy to assess atrophic gastritis (15–19). However, these tests have limitations in both accuracy and clinical application. IFA show high specificity (95–100%) for AIG and PA but suffer from poor sensitivity (approximately 27%) (20). PCA offer higher sensitivity (81%) but reduced specificity (90%). Combining both antibody tests improves overall diagnostic accuracy to 60.6% sensitivity with maintained 95–100% specificity, reaching up to 73% sensitivity in patients with clinical PA (20). Added to this, many General Practitioners in the United Kingdom (UK) have not adopted these extensive testing approaches (21). The inconsistent diagnostic outcomes are compounded by antibody testing’s variable performance at different disease stages (13, 15, 20, 22, 23).

Critically, patients with and without definitive PA confirmation face substantial barriers to accessing adequate treatment. The Pernicious Anaemia Society (PAS), a UK-based patient advocacy group, conducted two surveys (2012 and 2022) aimed at better understanding patient experiences and needs (24, 25). Previous analysis of diagnostic testing in PAS membership highlights diagnostic uncertainty, which directly impacts treatment access and QoL (24). Recent evidence also shows that patients whose injections were suspended or switched to oral supplementation during the COVID-19 pandemic reported worsening symptoms and reduced wellbeing compared to those who maintained their usual injection regimens, or those that were able to optimize their treatment frequency (26). Furthermore, a cross-sectional study found that 4 out of 10 patients with B_12_ deficiency reported self-injecting B_12_ obtained outside the National Health Service (NHS), primarily due to difficulty accessing treatment in primary care (27). A review from the UK’s National Institute for Health and Care Excellence (NICE) acknowledges this treatment gap, noting that intramuscular B_12_ is generally reserved for confirmed malabsorption cases, creating uncertainty when IFA are negative despite clinical B_12_ deficiency (28). This gatekeeping based on serological results rather than symptom burden means that treatment access is determined by diagnostic classification rather than clinical need, perpetuating a one-size-fits-all approach that may leave many patients undertreated (24).

We propose that clinically relevant symptoms may be more important than diagnostic labels, and that treatment should be guided by symptom control rather than diagnostic category. In line with this, the 2012 PAS survey, the dataset analyzed in the present study, found that while some patients respond well to the standard UK regimen, >60% of PAS members are dissatisfied with their treatment frequency and have trouble in maintaining an acceptable QoL. Traditional diagnostic categories might not adequately capture these differences, in response to standardized treatment, possibly explaining why they fail to satisfy many patients’ needs. Updated NICE guidelines acknowledge this by recommending that patients receive intramuscular hydroxocobalamin injections titrated to symptom severity or response (28–30). However, this recommendation is qualified by the requirement to prescribe within the medication’s licensed dosing schedule, which in practice may lead clinicians to default to standardized two- to three-monthly intervals regardless of individual symptom burden. Improving the quality of care for chronic, life-long conditions like PA therefore requires systematic changes guided by research advances and frameworks such as the chronic care model (CCM) (31). This advocates for delivery system restructure within clinical information systems to track patient outcomes, and self-management support to empower patients as partners in their care (31). Identifying symptom subtypes within this heterogeneous population is a critical step toward enabling such individualized approaches. This is particularly relevant given that the 2024 NICE guidelines, while recommending symptom-titrated treatment, describe symptoms in vague terms and provide limited guidance for patients who presenting with B_12_ deficiency without definitive PA confirmation. Although these data were collected in 2010–2012, our analytic objective, characterizing how symptoms co-occur to form distinct burden subtypes, reflects the underlying pathophysiology of B_12_ and PA, which has not changed over the intervening period.

Addressing both the NICE implementation gap and the broader challenge of tailoring treatment to diagnostic uncertainty requires first identifying which patient subgroups exist and what their differential needs are; the foundation of the CCM-informed, personalized care. Our primary hypothesis is that patients experiencing B_12_-related disorders comprise distinct subgroups characterized by their symptom presentations, and that these subgroups extend beyond the traditional diagnostic boundary separating PA from B_12_ deficiency of uncertain etiology. Our secondary hypothesis is that these symptom-defined subgroups reveal differential treatment responses and satisfaction levels, with symptom severity and pattern serving as stronger predictors of outcomes than a clinical diagnosis.

## Methods

### Schematic overview of survey program

Participant flow though the survey program is shown in Figure 1, from initial invitation of PAS members to the final analytic sample. The resulting analytic sample forms the basis for the symptom-subtyping analysis presented in this study. The survey was distributed to PAS members online and by post, with steps taken to prevent duplicate submission and to ensure data completeness prior to analysis; full detail on survey administration is provided in Survey procedures. Participation was voluntary, and completion of the survey was taken to indicate willingness to participate; the NHS Research Ethics Committee (NRES) determined that formal ethical approval was not required, as the study was classified as a service evaluation audit rather than clinical research (32).

**Figure 1:**
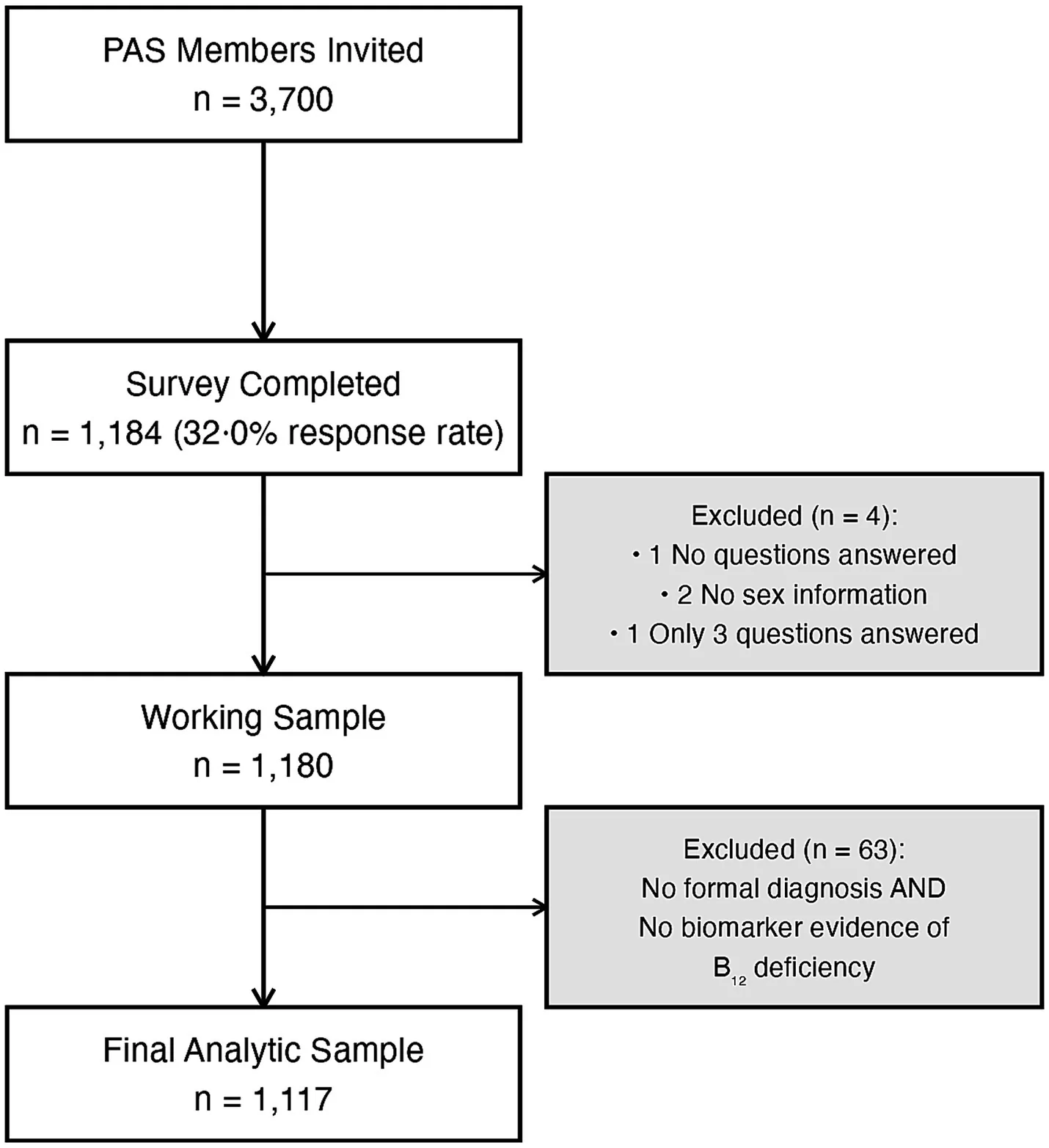
Study flow diagram. The flow chart illustrates participant recruitment and inclusion. From the 3,700 Pernicious Anaemia Society (PAS) members invited, 1,184 completed the survey (32.0% response rate). Four participants were excluded due to insufficient data (one provided no responses, two did not provide sex information, and one answered only three questions), yielding a working sample of 1,180. A further 63 participants were excluded who reported they had neither a formal diagnosis of B_12_ deficiency or pernicious anemia nor reported biomarker evidence of B_12_ deficiency (low serum B_12_, positive intrinsic factor antibody, or abnormal gastroscopy), resulting in a final analytic sample of *N* = 1,117.

### Study design

In this cross-sectional retrospective secondary analysis, we applied latent class analysis (LCA), a person-centered statistical approach that identifies unobserved subgroups within populations, to survey data collected by the PAS from their patient cohort (Supplementary Table 1) (24). Unlike previous descriptive studies, this approach identifies distinct symptom patterns and assesses their agreement with current diagnostic categories (25, 33). We examined relationships between symptom profiles, demographic characteristics, pre-diagnosis symptom duration, and treatment satisfaction across patients with B_12_-related disorders, independent of formal diagnostic classification, to evaluate if symptom burden provides greater guidance for treatment decisions compared to diagnostic status. This study follows the Strengthening the Reporting of Observational Studies in Epidemiology (STROBE) guidelines for cross-sectional studies, a checklist-based framework designed to promote transparent and complete reporting in observational research; we followed it to ensure our methods and results could be evaluated and reproduced with the same rigor expected of prospectively designed studies (32, 34).

### Participants

All PAS members (*n* = 3,700) were deemed eligible; eligibility was defined by PAS membership itself, rather than by any additional clinical criteria such as a confirmed diagnosis or biomarker status. This reflects the PAS’s role on supporting individuals across the full spectrum of B_12_-related diagnostic uncertainty, consistent with this study’s rationale for examining symptom burden independent of formal diagnostic classification. No exclusion criteria were applied at the point of invitation. New members who joined the PAS during the 15-month collection period were also offered the opportunity to complete the survey, on the same basis as existing members. The survey was made available online through the PAS website,^1^ and paper copies were mailed to members without Internet access. Exclusion criteria applied during subsequent analysis, and the resulting pathway from invited members to the final analytic sample of 1,117, are reported in the Results (Figure 1).

### Survey procedures

The survey was administered online via PAS website, where members completed it after logging into their account; to prevent duplicate submissions, members were not prompted to complete the survey again on subsequent logins. Members without internet access were mailed a paper copy to complete and return. All responses, whether submitted online or by post, were collated into a single electronic dataset. Data were collected over a 15-month period between August 2010 and November 2012 using a comprehensive four-section survey instrument. The survey collected: (a) demographic factors such as sex (male, female), age at diagnosis, and family history of PA; (b) symptoms experienced prior to diagnosis across seven physiological domains: general, neurological, respiratory, cardiovascular, gastrointestinal, genitourinary, and emotional; (c) coexisting medical conditions across eight areas: general, neurological, dermatologic, cardiovascular, musculoskeletal, endocrine, gastrointestinal, and emotional; and (d) diagnostics including biomarkers and endoscopy results, treatment type and frequency, and satisfaction with treatment. All symptom and disease variables were binary (presence/absence). Participants were specifically asked to report symptoms experienced prior to diagnosis rather than current symptoms, which helped to focus this analysis on symptom descriptions for use in a diagnostic and treatment management model. Prior to analysis, responses were screened for completeness; four participants who provided no or minimal usable data were excluded.

### Statistical analysis

A missing data pattern analysis was conducted to assess potential non-response bias; complete data were available for all 46 LCA indicator variables, with missing data observed only in post-hoc outcome variables (Supplementary Figure 1). To assess potential recall bias associated with retrospective symptom reporting, Spearman rank correlations were used to examine the relationship between duration of symptoms before diagnosis and individual symptom endorsement across all 35 symptom indicators (35).

### Latent class analysis

We employed LCA to determine distinct symptom profiles within the study population. LCA is a person-centered statistical approach that identifies unobserved subgroups (latent classes) within a population based on response patterns across multiple observed variables (36). We selected symptoms and comorbid conditions as observed indicators for the LCA based on prevalence within our sample. Following the methodological recommendations of Sinha et al., we included only indicators with a prevalence of at least 10% (37). This threshold helps prevent the identification of latent classes that are exclusively based on rare indicators, which result in the classes driven by rare responses rather than meaningful population heterogeneity. This resulted in 46 indicators comprising three demographic indicators, 35 symptoms, and eight comorbid condition indicators representing the most common manifestations of B_12_ deficiency in our population.

Models with 2–10 classes were estimated and compared using multiple fit indices including Akaike Information Criterion (AIC), Bayesian Information Criterion (BIC), and sample-size adjusted BIC (aBIC), along with entropy and Lo-Mendell-Rubin adjusted likelihood ratio tests (LMR-LRT). Lower information criterion values (AIC, BIC, aBIC) indicate better model fit, with BIC typically given greater weight as it penalizes model complexity more strongly than AIC and aBIC, reducing the risk of overfitting. Higher entropy values (closer to 1.0) indicate better classification quality, and a significant LMR-LRT *p*-value indicates that the k-class model fits significantly better than the (k-1)-class model. Model selection involved visual examination of elbow plots for all information criteria to identify where diminishing returns in fit improvement occurred.

### *Post-hoc* analysis

To examine the relationship between symptom burden and autoimmune etiology, IFA test results were analyzed across the three latent class subtypes. Only participants with a reported IFA result (either positive or negative) were included in the analysis (*n* = 222, 19.9% of the analytic sample). A chi-square test was used to examine whether IFA positivity differed significantly across the three symptom burden subtypes.

Associations between latent class membership and two patient characteristics were examined. Duration of symptoms before receiving a diagnosis was collected across 11 ordered categories: 1-week, 2-weeks, 1-month, 2-months, 3-months, 6-months, 1-year, 2-years, 5-years, 10-years, and 10+ years. These timeframes were selected based on their clinical relevance. For visualization, duration categories were collapsed into three broader, clinically relevant groups: less than 6 months, 6 months to 2 years, and more than 2 years; the full 11-category distribution was analyzed separately. Chi-square analysis with Cramér’s V effect size tested whether symptom duration differed across symptom burden subtypes for both collapsed and full category schemes. Age when symptoms first started was analyzed as a categorical variable in nine ranges (0–10, 11–20, 21–30, 31–40, 41–50, 51–60, 61–70, 71–80, 81–90) and evaluated using chi-square analysis to test for associations with symptom burden subtype. To identify which specific age and subtype combinations drove any observed associations, standardized residuals were calculated; absolute values above 1.96, corresponding to *p* < 0.05 under the standard normal distribution, were treated as statistically significant. Participants with missing data for age when symptoms commenced were excluded listwise from this analysis (*n* = 1,013, 90.7% of the analytic sample).

Treatment frequency and satisfaction rates were examined across symptom burden subtypes. Frequency categories were based on standard and self-directed administration schedules for B_12_ replacement therapy and collapsed where appropriate: weekly or more (including daily, more than once a day, and weekly), monthly, two-monthly, three-monthly, other, or no treatment. Participants with missing treatment frequency data (*n* = 155, 13.9% of analytic sample) or missing satisfaction responses (*n* = 37) were excluded listwise. Fisher’s exact tests with 10,000 Monte Carlo simulations compared satisfaction rates for each frequency category against three-monthly injections as the reference category. Free-text responses in the “Other” category were categorized into five groups based on injection interval: loading phase, very frequent (every 2 weeks or less), extended standard (3–11 weeks), extended interval (12 weeks or more), and no treatment or pre-treatment.

All analyzes were conducted using Mplus Version 8 for LCA and R Statistical Software (version 4.0.3; R Core Team 2021) for descriptive statistics and post-hoc analyzes (38, 39).

## Results

Of the 3,700 PAS members invited to participate, 1,184 completed the survey (response rate, 32.0%). Four respondents were excluded from analysis: one did not answer any questions, two did not provide sex information, and one answered only three questions, yielding a working sample of 1,180. A further 63 participants were excluded; they reported neither a formal diagnosis of B_12_ deficiency or PA nor biomarker evidence of B_12_ deficiency (low serum B_12_, positive IFA, or abnormal gastroscopy). This resulted in a final analytic sample of 1,117 participants (Figure 1). The analytic included PAS members across the diagnostic spectrum, encompassing those who reported a formal PA diagnosis and B_12_ deficiency without PA diagnosis.

Our LCA comprised data on 1,117 participants from the PAS, with age when symptoms first started ranging from 0 to 90 years. Most participants were female (*n* = 911, 81.6%) and a majority of participants reported a formal diagnosis of PA (*n* = 853, 76.4%), while 243 (21.7%) reported B_12_ deficiency without PA diagnosis. A further 13 (1.2%) reported no formal diagnosis but were retained based on reported biomarker evidence of B_12_ deficiency. Complete data were available for all 46 LCA indicator variables across the full analytic sample. Missing data were only observed in post-hoc outcome variables: age when symptoms first started (9.3%), duration of symptoms before diagnosis (6.4%), and treatment satisfaction (8.1%; Supplementary Figure 1). While 33 of 35 symptoms (94.3%) showed statistically significant correlations with symptom duration, the magnitude of these correlations was weak (ranging from −0.032 to 0.205; Supplementary Figure 2, Supplementary Table 2) (35).

We tested latent class models with 2–10 classes to identify distinct symptom subtypes among participants with B_12_ deficiency and/or PA (Supplementary Figure 3, Supplementary Table 3). The 3-class solution emerged as the best fit and most parsimonious based on convergence of fit indices, LMR-LRT significance, and adequate class sizes. This model showed significant improvement over the 2-class solution across all information criteria, with lower values for AIC (55,866 vs. 56,733), BIC (56,568 vs. 57,200), and sample-size adjusted BIC (aBIC: 56,123 vs. 56,904; Supplementary Figure 3, Supplementary Table 3). While AIC and aBIC continued to decrease with additional classes, BIC showed an inflection point at the 3-class solution before increases became more gradual, suggesting diminishing returns in model fit beyond this point. The 3-class model fit was significantly better than the 2-class solution (*p* < 0.0001) as confirmed with the LMR-LRT. Importantly, the LMR-LRT test for the 4-class solution was non-significant (*p* = 0.51), indicating no statistically meaningful improvement over the 3-class model despite the slightly lower BIC. Additionally, the 3-class solution showed high entropy (0.86), indicating clear separation between classes and robust classification of participants. The three identified subtypes are described in detail below, labeled High Burden, Moderate Burden, and Low Burden based on cumulative symptom prevalence across domains.

The High Burden subtype (*n* = 334, 30.0%) was characterized by the highest symptom burden across all domains (Table 1), exhibiting fatigue (99.4%) and waking up tired (98.5%), accompanied by cognitive dysfunction, including poor concentration (98.5%), memory loss (97.9%), and confusion (93.7%). Neurological manifestations were prominent, with high rates of clumsiness (95.5%), paresthesia (86.2%), and dizziness (91.0%), together with significant mood disturbances, including irritability (94.9%), and impatience (90.7%). Notably, this subtype had the highest rate of suicidal thoughts (41.6%) and highest prevalence of affected grandparents (18.0%). Comorbid conditions were common across multiple systems, including cardiovascular (arrhythmia 37.4%), endocrine (hypothyroidism 24.3%), dermatological conditions (vitiligo 21.6%; psoriasis/eczema/acne 38.0%), and general medical conditions (tinnitus 55.4%; folic acid deficiency 32.2%). Depression, classified as a distinct emotional condition rather than a symptom, was notably prevalent in this subtype (69.8%).

**Table 1:** Demographic characteristics, symptoms, and comorbidities across latent classes of vitamin B_12_ deficiency (*N* = 1,117).

No. (%)					
Characteristic	Latent class analysis group				*p* value
	Total (*N* = 1,117)	HB (*n* = 334)	MB (*n* = 613)	LB (*n* = 170)	
Demographic					
Sex (F)	911 (81.6%)	307 (91.9%)	495 (80.8%)	109 (64.1%)	<0.0001
Grandparent	174 (15.6%)	60 (18.0%)	98 (16.0%)	16 (9.4%)	0.04
Parent	165 (14.8%)	56 (16.8%)	85 (13.9%)	24 (14.1%)	0.51
General symptoms					
Tiredness	1,063 (95.2%)	332 (99.4%)	601 (98.0%)	130 (76.5%)	<0.0001
Hair loss or graying	343 (30.7%)	168 (50.3%)	162 (26.4%)	13 (7.6%)	<0.0001
Brittle nails	412 (36.9%)	170 (50.9%)	209 (34.1%)	33 (19.4%)	<0.0001
Brittle nails with ridges	540 (48.3%)	231 (69.2%)	265 (43.2%)	44 (25.9%)	<0.0001
Weight loss	237 (21.2%)	96 (28.7%)	122 (19.9%)	19 (11.2%)	<0.0001
Wake up tired	975 (87.3%)	329 (98.5%)	550 (89.7%)	96 (56.5%)	<0.0001
Dry skin	655 (58.6%)	274 (82.0%)	335 (54.6%)	46 (27.1%)	<0.0001
Flushes fever	468 (41.9%)	235 (70.4%)	214 (34.9%)	19 (11.2%)	<0.0001
Glossitis	378 (33.8%)	185 (55.4%)	170 (27.7%)	23 (13.5%)	<0.0001
Neurological symptoms					
Poor concentration	851 (76.2%)	329 (98.5%)	467 (76.2%)	55 (32.4%)	<0.0001
Pins and needles (paresthesia)	743 (66.5%)	290 (86.1%)	387 (63.2%)	67 (39.4%)	<0.0001
Confusion	685 (61.3%)	313 (93.7%)	347 (56.6%)	25 (14.7%)	<0.0001
Nominal aphasia	563 (50.4%)	286 (85.6%)	249 (40.6%)	28 (16.5%)	<0.0001
Clumsiness	734 (65.7%)	319 (95.5%)	388 (63.3%)	27 (15.9%)	<0.0001
Headaches	560 (50.1%)	246 (73.7%)	289 (47.1%)	25 (14.7%)	<0.0001
Grierson Gopalan Syndrome (burning legs and feet)	383 (34.3%)	195 (58.4%)	156 (25.4%)	32 (18.8%)	<0.0001
Forgetfulness/memory loss	884 (79.1%)	327 (97.9%)	488 (79.6%)	69 (40.6%)	<0.0001
Poor sleep	717 (64.2%)	289 (86.5%)	372 (60.7%)	56 (32.9%)	<0.0001
Dizziness	649 (58.1%)	304 (91.0%)	320 (52.2%)	25 (14.7%)	<0.0001
Shoulder bumps	554 (49.6%)	282 (84.4%)	259 (42.3%)	13 (7.6%)	<0.0001
Cannot stand eyes closed	374 (33.5%)	218 (65.3%)	144 (23.5%)	12 (7.1%)	<0.0001
Vertigo	360 (32.2%)	205 (61.4%)	143 (23.3%)	12 (7.1%)	<0.0001
Respiratory symptoms					
Shortness of breath (the sighs)	808 (72.3%)	309 (92.5%)	438 (71.5%)	61 (35.9%)	<0.0001
Cardiovascular symptoms					
Palpitations	618 (55.3%)	269 (80.5%)	321 (52.4%)	28 (16.5%)	<0.0001
Gastrointestinal symptoms					
Indigestion	481 (43.1%)	234 (70.1%)	223 (36.4%)	24 (14.1%)	<0.0001
Loss of appetite	303 (27.1%)	143 (42.8%)	137 (22.3%)	23 (13.5%)	<0.0001
Taste loss or change	290 (26.0%)	163 (48.4%)	116 (18.9%)	11 (6.5%)	<0.0001
Stomach cramps	443 (39.7%)	220 (65.9%)	207 (33.8%)	16 (9.4%)	<0.0001
Diarrhea	652 (58.4%)	269 (80.5%)	345 (56.3%)	38 (22.4%)	<0.0001
Diarrhea alternating with constipation	457 (40.9%)	219 (65.6%)	221 (36.1%)	17 (10.0%)	<0.0001
Genitourinary symptoms					
Bladder/kidney infections	231 (20.7%)	112 (33.5%)	111 (18.1%)	8 (4.7%)	<0.0001
Emotional symptoms					
Irritability/frustration	848 (75.9%)	317 (94.9%)	473 (77.2%)	57 (34.1%)	<0.0001
Impatience/desire isolation	721 (64.5%)	303 (90.7%)	382 (62.3%)	36 (21.2%)	<0.0001
Sudden mood swings	641 (57.4%)	286 (85.6%)	332 (54.2%)	23 (13.5%)	<0.0001
Suicidal thoughts	244 (21.8%)	139 (41.6%)	103 (16.8%)	2 (1.2%)	<0.0001
General medical conditions					
Tinnitus	389 (34.8%)	185 (55.4%)	172 (28.1%)	32 (18.8%)	<0.0001
Folic acid deficiency	256 (22.9%)	112 (32.2%)	129 (21.0%)	16 (9.4%)	<0.0001
Dermatological conditions					
Acne rosacea	172 (15.4%)	74 (22.2%)	84 (13.7%)	14 (8.2%)	<0.0001
Psoriasis/eczema/acne	315 (28.2%)	127 (38.0%)	168 (27.4%)	20 (11.8%)	<0.0001
Vitiligo	156 (14.0%)	72 (21.6%)	72 (11.7%)	12 (7.1%)	<0.0001
Cardiovascular conditions					
Arrhythmia	246 (22.0%)	125 (37.4%)	104 (17.0%)	17 (10.0%)	<0.0001
Endocrine conditions					
Hypothyroidism	223 (20.0%)	81 (24.3%)	126 (20.6%)	16 (9.4%)	<0.001
Emotional conditions					
Depression	516 (46.2%)	233 (69.8%)	259 (42.2%)	24 (14.1%)	<0.0001

This table presents the distribution of demographic characteristics, clinical symptoms, and comorbid conditions across the three latent classes identified through latent class analysis. The High Burden subtype (*n* = 334) demonstrates the highest symptom prevalence across all domains, the Moderate Burden subtype (*n* = 613) exhibits intermediate symptom prevalence, and the Low Burden subtype (*n* = 170) shows substantially lower symptom prevalence. Values represent number (percentage) of participants within each class reporting the presence of each indicator. *p*-values derive from chi-square tests comparing prevalence across classes. Notably, 45 of 46 indicators (97.8%) showed statistically significant differences across classes (*p* < 0.05), with only parental history of pernicious anemia (*p* = 0.51) showing similar distribution across all three groups. HB, High Burden; MB, Moderate Burden; LB, Low Burden.

The Moderate Burden subtype (*n* = 613, 54.9%) presented with intermediate symptom prevalence (Table 1). Fatigue in this subtype remained high (98.0%), but cognitive symptoms were less prevalent, with lower rates of memory loss (79.6%), poor concentration (76.2%), and confusion (56.6%). Neurological manifestations followed a similar pattern, with moderate rates of paresthesia (63.3%) and clumsiness (63.3%). Mood disturbances were present but less pronounced than the High Burden subtype, including suicidal thoughts in 16.8%. Depression was reported by 42.3% of this subtype, reflecting a similar distinct condition pattern as observed in the High Burden class.

The Low Burden subtype (*n* = 170, 15.2%) exhibited a markedly reduced symptom prevalence (Table 1) and showed a lower female predominance (64.1% compared to 81.0 and 91.9% for Moderate and High Burden subtypes) than the other subtypes. Fatigue, while still present (76.5%), was significantly less prevalent than in other subtypes. Cognitive dysfunction was minimal, with lower rates of memory loss (40.6%), and confusion (14.7%). Neurological symptoms were rare, with minimal clumsiness (15.9%) and dizziness (14.7%). This subtype had minimal mental health impact, with depression affecting only 14.1% and suicidal thoughts in just 1.2%.

IFA test results were available for 222 participants (19.9% of the analytic sample), limiting the interpretability of antibody-based comparisons across subtypes. Among those tested, positive IFA results were similarly distributed across subtypes: 49.4% in the High Burden subtype, 56.2% in the Moderate Burden subtype, and 57.1% in the Low Burden subtype (*χ*^2^ = 1.04, df = 2, *p* = 0.593; Table 2).

**Table 2:** Intrinsic factor antibody (IFA) test results across latent classes (*n* = 222 tested, 19.9% of analytic sample).

*N* (%)				
Class subtype	IFA positive	IFA negative	Total tested	% Positive
High burden	44	45	89	49.4%
Moderate burden	59	46	105	56.2%
Low burden	16	12	28	57.1%

The proportion of positive IFA results is reported descriptively for each symptom burden subtype. A chi-square test comparing IFA positivity across the three subtypes showed no significant difference (*χ*^2^ = 1.04, df = 2, *p* = 0.593), indicating that the likelihood of a positive IFA result was similar regardless of symptom burden subtype. These findings suggest that autoimmunity etiology, as measured by IFA, does not differentiate symptom burden subtypes. Interpretation should be made with caution given the poor sensitivity of IFA (approximately 27%); a negative result does not exclude pernicious anemia.

Age at symptom onset differed significantly across subtypes (*χ*^2^ = 37.07, df = 16, *p* = 0.002, Cramér’s *V* = 0.14), with the Low Burden subtype showing overrepresentation in older age groups, particularly ages 71–80 (*z* = 3.54) and 61–70 (*z* = 2.52). The High Burden subtype showed overrepresentation in the 31–40 age group (*z* = 2.04) and underrepresentation in the 71–80 age group (*z* = −2.50). The Moderate Burden subtype demonstrated a more balanced age distribution closely matching the overall sample (Figure 2, Supplementary Table 4; *n* = 1,013).

**Figure 2:**
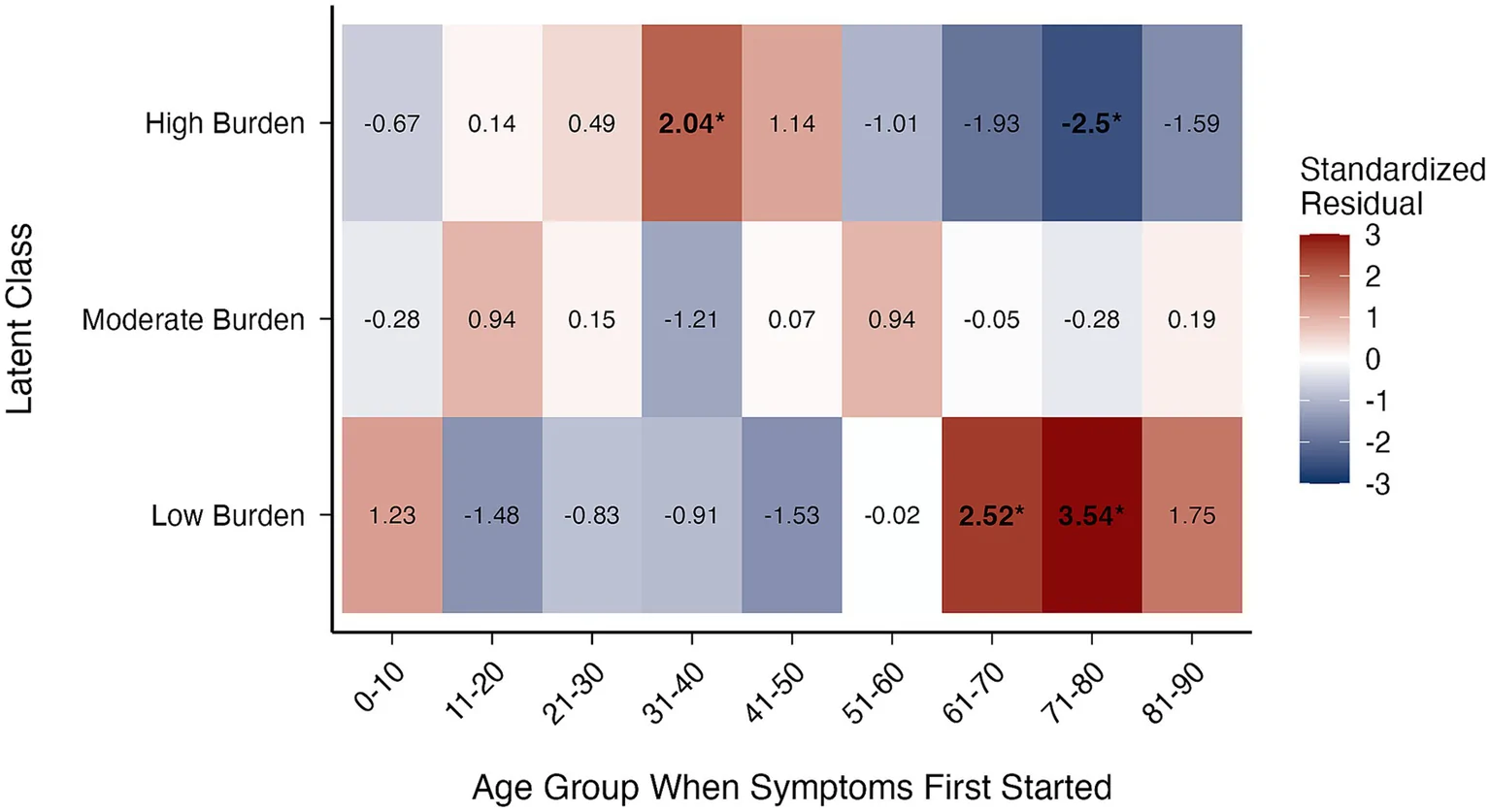
Standardized residuals from chi-squared analysis of age when symptom first started by symptom burden subtype (High Burden *n* = 295, Moderate Burden *n* = 563, Low Burden *n* = 155, total *n* = 1,013). Standardized residuals indicate the degree to which observed cell counts deviate from expected counts under independence. Positive values (red) indicate more observations than expected; negative values (blue) indicate fewer. Bold values with asterisks (^*^) denote statistically significant overrepresentations (|*z*| > 1.96, *p* < 0.05). The Low Burden subtype shows significant overrepresentation in the 61–70 (z = 2.52) and 71–80 (*z* = 3.54) age groups. The High Burden subtype shows significant overrepresentation in the 31–40 age group (*z* = 2.04) and significant underrepresentation in the 71–80 age group (*z* = −2.50), suggesting that younger age of symptom onset is associated with higher symptom burden. Data were available for 1,013 of 1,117 participants (90.7%); participants with missing age data were excluded listwise.

Symptom duration pre-diagnosis varied significantly between subtypes (*χ*^2^ = 115.47, df = 4, *p* < 0.001, Cramér’s *V* = 0.23; *n* = 1,046). The High Burden subtype experienced longer pre-diagnosis symptom periods, with 56.9% reporting symptoms of more than 2 years before diagnosis, compared with 40.6% in the Moderate Burden subtype and dropping to 19.2% in the Low Burden subtype. Only 2.5% of the High Burden subtype received a diagnosis within 6 months of symptom onset, compared to 4.4% in the Moderate Burden subtype and 21.8% in the Low Burden subtype (Figure 3; full 11-category distribution in Supplementary Figure 4).

**Figure 3:**
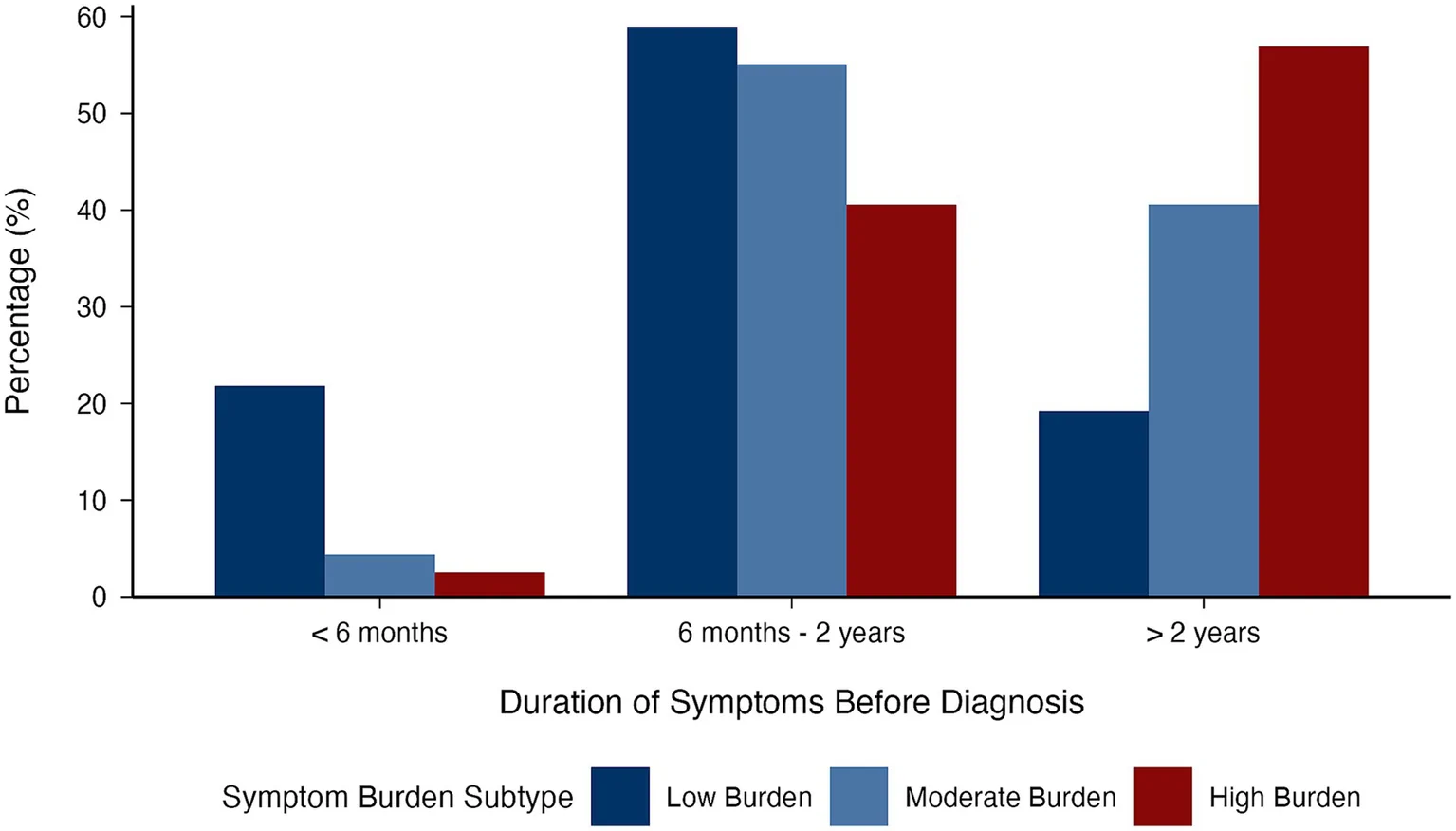
Duration of symptoms before diagnosis across symptom burden subtypes (High Burden *n* = 318, Moderate Burden *n* = 572, Low Burden *n* = 156, total *n* = 1,046). Values represent number and percentage of patients within each symptom burden subtype reporting each duration category. Duration categories are collapsed into three groups: less than 6 months, 6 months to 2 years, and more than 2 years (see Supplementary Figure S4 for the full 11-category distribution). Chi-square analysis revealed highly significant differences in symptom duration before diagnosis (*χ*^2^ = 115.47, df = 4, *p* < 0.001, Cramér’s *V* = 0.23), with High Burden subtype showing the longest pre-diagnosis periods compared to Moderate and Low Burden subtypes. Participants with missing data for symptom duration were excluded listwise (71 of 1,117 participants).

Three-monthly injections showed consistently low satisfaction across all subtypes (High Burden: 8.5%; Moderate Burden: 23.7%; Low Burden: 30.7%; Table 3). In the High Burden and Moderate Burden subtypes, all frequencies except two-monthly were associated with significantly higher satisfaction than three-monthly injections (all *p* < 0.001). Two-monthly injections approached but did not reach significance in the High Burden subtype (*p* = 0.063), while all frequencies including the two-monthly reached significance in the Moderate Burden subtype (*p* < 0.001). The Low Burden subtype showed significance in the monthly (*p* = 0.037) and “Other” (*p* = 0.002). The “Other” category showed the highest satisfaction rates across all subtypes (High Burden: 45.9%; Moderate Burden: 58.8%; Low Burden: 73.3%; Table 3, Supplementary Table 5).

**Table 3:** Treatment frequency and satisfaction rates across symptom burden subtypes of vitamin B_12_ deficiency (*N* = 1,117).

Treatment frequency	High burden		Moderate burden		Low burden		Overall	
	*n*	% Satisfied	*n*	% Satisfied	*n*	% Satisfied	*n*	% Satisfied
Weekly or more	25 (9.1%)	10 (40.0%)^***^	33 (6.4%)	18 (54.5%)^***^	8 (5.8%)	5 (62.5%)	66 (7.1%)	33 (50.0%)
Monthly	52 (18.8%)	18 (34.6%)^***^	66 (12.9%)	32 (48.5%)^***^	19 (13.8%)	11 (57.9%)^*^	137 (14.8%)	61 (44.5%)
Two monthly	44 (15.9%)	9 (20.5%)	84 (16.4%)	40 (47.6%)^***^	21 (15.2%)	10 (47.6%)	149 (16.1%)	59 (39.6%)
Three monthly	118 (42.8%)	10 (8.5%)	262 (51.1%)	62 (23.7%)	75 (54.3%)	23 (30.7%)	455 (49.1%)	95 (20.9%)
Other	37 (13.4%)	17 (45.9%)^***^	68 (13.3%)	40 (58.8%)^***^	15 (10.9%)	11 (73.3%)^**^	120 (12.9%)	68 (56.7%)

*n* (%) represents number of participants with a recorded treatment frequency and satisfaction response within each subtype. 155 of 1,117 participants (13.9%) had missing treatment frequency data and were excluded. Satisfaction percentages are calculated among those who responded Yes or No only. Three-monthly injections represent the standard NHS treatment frequency and serve as the reference category. ^*^p < 0.05, ^**^p < 0.01, ^***^p < 0.001 vs. three-monthly (Fisher’s exact test with 10,000 Monte Carlo simulations). LCA, latent class analysis; NHS, National Health Service; PA, pernicious anemia.

## Discussion

Our study identified three distinct symptom-based subtypes within a predominantly PA confirmed population (76.4% with a formal PA diagnosis), including patients with B_12_ deficiency of uncertain etiology (21.8%). These subtypes, which we designate as High, Moderate, and Low Burden, were defined by cumulative symptom prevalence across multiple domains rather than diagnostic classification, addressing our objective to characterize clinical heterogeneity in this population. Each subtype demonstrated distinctive symptom profiles, demographic features, and treatment needs, supporting our hypothesis that symptom patterns, rather than diagnostic labels based on serological markers, better guide personalized management approaches (Figure 4). This represents the first systematic application of person-centered methodology in this patient population, extending beyond current literature which focuses primarily on cataloging symptoms rather than identifying subtypes (15, 17, 25, 33).

**Figure 4:**
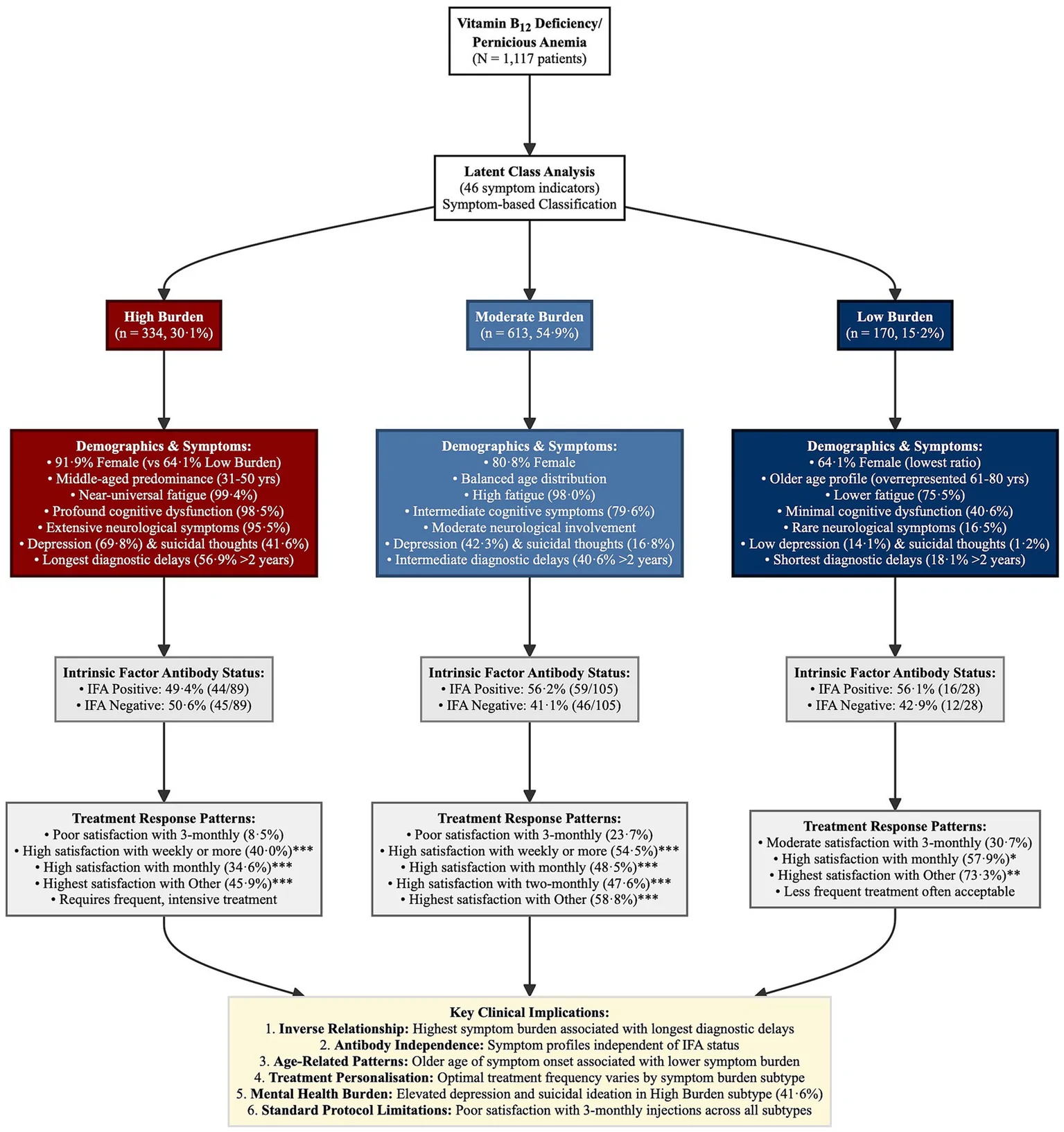
Latent class analysis of vitamin B_12_ deficiency/pernicious anemia symptom profiles and clinical characteristics. Study findings showing the three-subtype solution derived from latent class analysis of 46 indicators in 1,117 participants with vitamin B_12_ deficiency and/or pernicious anemia. The analysis identified three distinct symptom-based subtypes: High Burden, *n* = 334 (29.9%), characterized by near-universal fatigue, profound cognitive dysfunction, and extensive neurological symptoms with the highest female predominance and longest diagnostic delays; Moderate Burden, *n* = 613 (54.9%), showing intermediate symptom severity across all domains; and Low Burden, *n* = 170 (15.2%), presenting with limited symptoms, older age profile, and shortest diagnostic delays. Intrinsic factor antibody (IFA) status did not significantly differentiate between subtypes (*χ*^2^ = 1.04, df = 2, *p* = 0.593; *n* = 222 tested), while treatment response patterns varied by symptom burden subtype, with the High Burden subtype showing low satisfaction with three-monthly injection protocols and higher satisfaction with more frequent administration. Key clinical implications include the inverse relationship between symptom burden and diagnostic delay, the inadequacy of current standard treatment protocols across all subtypes, and the critical need for personalized, symptom-guided treatment approaches rather than antibody classification.

Previous literature describes both B_12_ deficiency and PA as clinically heterogenous, spanning neurological, hematological, psychiatric, and gastrointestinal manifestations (7). Our analysis reveals that seemingly dissimilar symptoms cluster into distinct patterns giving three subtypes defined by cumulative symptom prevalence rather than individual symptom intensity. Whether these subtypes represent etiologically distinct phenotypes or severity strata along single disease continuum remains an open question for future studies. However, several patterns suggest these may be different disease processes rather than simply varying severity: the non-linear gradient of suicidal thoughts (41.6% in High Burden vs. 16.8% in Moderate Burden vs. 1.2% in Low Burden), the older age profile of the Low Burden subtype, and differential multigenerational family history patterns are not straightforwardly explained by a simple severity spectrum. Prospective studies incorporating biomarkers, genetic profiling, and longitudinal symptom assessment will be necessary to determine whether these subtypes reflect different disease mechanisms or clinically meaningful points along a severity spectrum. This distinction has important implications for diagnostic classification and treatment stratification.

The High Burden subtype presented with overwhelming multisystem involvement, near-universal fatigue, waking tired, profound cognitive dysfunction including memory loss, poor concentration, and confusion, alongside prominent neurological manifestations with high rates of clumsiness, dizziness, and paresthesia. The Moderate Burden subtype, representing the largest group, demonstrated intermediate symptom burden; fatigue remained prevalent, but cognitive, neurological, and psychological symptoms were significantly less pronounced. The Low Burden subtype was an attenuated phenotype with a lower symptom burden across all domains. The shifting female-to-male ratio across subtypes (High Burden 13:1, Moderate Burden 5:1, Low Burden 3:1) also warrants follow-up, though these ratios may reflect PAS membership composition and volunteer bias rather than true population distributions.

The absence of association between IFA status and symptom burden (49.4, 56.2, 57.1%) must be interpreted alongside the well-documented technical limitations of IFA testing (20). The approximately equal distribution of positive and negative results across all burden classes likely reflects the poor sensitivity of current antibody assays rather than meaningful biological distinctions. IFA testing suffers from substantial technical variability, with low sensitivity (as low as 27%) despite high specificity (>80%), making it unreliable for differentiating patient subgroups or predicting clinical outcomes (20). This finding underscores that current serological markers are inadequate for stratifying patients or guiding treatment decisions. What remains clearer from our analysis is that symptom burden, independent of serological markers, strongly influences treatment needs and satisfaction, suggesting that clinical phenotyping may be a useful tool for development of more precise management plans.

While PA may take 10–12 years to progress from AIG to clinical presentation, it remains unknown whether diagnostic delays are associated with symptom burden (8, 40). Notably, we found an inverse relationship where individuals within the High Burden subtype, who are predominantly diagnosed before age 60, experienced significantly longer diagnostic delays (56.9% reporting more than 2 years before diagnosis) despite exhibiting widespread multisystem symptom profiles. This can be interpreted in several ways. First, lower symptom burden among older patients may reflect age-associated changes in gastric physiology, immune function, or nutrient absorption, possibly indicating slower disease progression and milder symptomatology burden; and diagnostic attribution bias, whereby symptoms in older patients are more readily ascribed to normal aging rather than investigated as potential B_12_ deficiency, potentially compounding delay recognition. Second, High Burden B_12_ deficiency in younger patients might represent a distinct, more aggressive phenotype, driven by stronger autoimmune and/or genetic factors (41, 42). The higher rates of multigenerational family history in the High Burden subtype support this interpretation, suggesting stronger genetic predisposition potentially linked to HLA-DRB1*03, HLA-DRB1*04, and other autoimmune susceptibility alleles (41). Beyond these interpretations, B_12_-dependent enzymatic pathways offer a plausible biological basis for symptom heterogeneity observed across subtypes. Deficiency in adenosyl-B_12_ leads to MMA accumulation, while methyl-B_12_ deficiency results in homocysteine elevation and impaired methylation affecting myelin synthesis and neurotransmitter production (18, 43). Whether differential disruption of these pathways underlies the symptom burden gradient remains to be established, though the neurological and neuropsychiatric manifestations of the High Burden subtype are consistent with this possibility (44). This subtype also had the highest rate of self-reported folic acid deficiency (33.2%), which, while not clinically verified in this study, raises the possibility that micronutrient deficiencies beyond B_12_ may contribute to symptom burden in this group (45, 46). However, folate supplementation in inadequately treated B_12_ deficiency may mask hematological signs of deficiency while accelerating neurological deterioration (47). Given the pronounced neurological burden already observed in this subtype, ensuring adequate B_12_ replacement before or alongside folate supplementation is essential (48, 49).

Across all three symptom subtypes, we identified significant associations between treatment frequency and satisfaction. The highest satisfaction rates were observed with alternative treatment approaches outside standard schedules (Other: 45.9–73.3%), followed by weekly or more frequent injections (40.0–62.5%), then monthly treatments (34.6–57.9%). Standard three-month injections showed consistently poor satisfaction across all subtypes (8.5–30.7% satisfaction), supporting the need for more symptom-tailored treatment protocols. Notably, the Low Burden subtype demonstrated significant satisfaction gains even at comparatively infrequent intervals (monthly and “other” frequencies), suggesting greater acceptability of less frequent treatment among individuals with milder symptom burden. Kornic et al. reported that more frequent injection intervals were associated with greater patient-reported symptom improvement (50). Our demonstration that optimal treatment frequency varies by symptom-burden subtype extends prior research by showing that symptom phenotypes are associated with differential treatment response patterns and provides supportive evidence for exploring personalized, symptom-based therapeutic approaches. Because treatment frequency was not randomly assigned, this association may also reflect confounding by indication or reverse causation. Participants with greater symptom burden may have sought or been prescribed more frequent injections, rather than frequency itself improving satisfaction. It should be noted, however, that neurological and psychiatric symptoms are known to show greater resistance to B_12_ supplementation than hematological manifestations, meaning that lower satisfaction in the High Burden subtype may partly reflect treatment-resistant symptomology rather than inadequate administration frequency alone (51). This limits causal interpretation of the satisfaction gradient and underscores the need for pragmatic trials examining biological response across symptom subtypes.

The mental health gradient across subtypes warrants particular attention. The High Burden subtype showed remarkably high rates of depression (69.8%) and suicidal ideation (41.6%), substantially exceeding both Moderate Burden (42.3%; 16.8%) and Low Burden (14.1%; 1.2%) subtypes. These observations likely reflect at least two complementary mechanisms. First, B_12_ deficiency creates direct biological vulnerability to psychiatric manifestations through impaired neurotransmitter synthesis and disrupted methylation pathways, effects that may be most pronounced in those with the greatest overall metabolic burden (47, 52, 53). Second, prolonged diagnostic delay imposed substantial situational and existential load on top of this biological vulnerability, encompassing feelings of medical invalidation, grief over lost functional years, and compounding social stressors such as occupational disruption and financial strain (54, 55). These mechanisms are not mutually exclusive, biological vulnerability and situational burden are likely to interact and amplify one another, particularly among those experiencing the longest delays. The predominantly female composition of the High Burden subtype (91.9%) adds a further dimension. Evidence documents that women are disproportionately subject to medical gaslighting, the systemic attribution of physical symptoms to psychological cause, which may deepen both diagnostic delay and psychological distress through cumulative invalidating clinical encounters (56). This pattern likely compounds the biological vulnerability and situational burden already experienced by this subtype, further contributing to the mental health gradient observed across subtypes. This suggests that High Burden B_12_ deficiency warrants integrated clinical attention addressing both underlying deficiency and its psychological sequelae, particularly given the high reported rates of suicidal ideation (57). Further research at the intersection of B_12_ deficiency and mental health, including neurobiological mechanisms and adjunctive psychiatric treatment approaches, is warranted.

Addressing the treatment frequency gradient and the mental health burden identified across subtypes requires systematic changes to clinical practice. This aligns with principles of the CCM, a framework stating that effective management of chronic conditions requires prepared, proactive healthcare teams working collaboratively with informed, engaged patients rather than reactive, crisis-driven care (31). The 2024 NICE guideline, recommends titrating intramuscular B_12_ to symptom severity or response rather than following standardized protocols, representing a significant policy shift toward this patient-centered model. However, the guidelines provide limited guidance on how clinicians should operationalize symptom titration in practice. Our identification of distinct symptom subtypes, with differential treatment needs, provides support for an evidence-based framework for operationalizing the NICE recommendations through CCM-informed approaches. For example, clinical information systems could track symptom profiles and treatment responses over time, delivery system redesign could enable flexible dosing protocols tailored to symptom subtype, and self-management support could help patients monitor their symptoms, self-adjust their injection frequency, and communicate changes to providers (30, 58). The CCM framework is particularly well-suited to this population given evidence that integrated management of depression alongside the underlying chronic condition produces better outcomes than treating each in isolation, an approach directly relevant to the High Burden subtype where biological and psychosocial contributors to depression likely to coexist.

This study has notable strengths. We used data-driven agnostic methods to identify latent subgroups and examined how symptom profiles predict treatment response, moving beyond the descriptive satisfaction rates or treatment preferences reported in previous research (24, 26, 50). By including patients across a diagnostic spectrum rather than restricting analysis to confirmed PA cases, we examined symptom patterns independent of diagnostic labels. Importantly, participants were asked to report symptoms experienced prior to diagnosis rather than current symptoms, anchoring the analysis to the pre-diagnostic presentation most relevant for informing clinical recognition and treatment stratification. Our comprehensive assessment of symptoms across multiple domains provides a holistic view of disease burden not captured by traditional clinical parameters. Additionally, the consistency of findings across different diagnostic categories and demographic subgroups enhances generalizability within similar patient advocacy populations.

Several limitations merit consideration. Our sample consisted exclusively of PAS members, who represent a highly engaged patient population that may differ systematically from broader B_12_-deficient populations, potentially overrepresenting those with High Burden symptoms or treatment dissatisfaction and limiting applicability to community-based clinical settings (24, 26, 50). The predominance of female participants (81.6%), and the limited ethnic diversity within PAS membership prevents examination of potential gender and ethnic disparities in PA prevalence or symptom severity, an important area for future investigation in more diverse populations.

The survey was administered between 2010 and 2012, prior to the 2024 revision of the NICE guidelines on B_12_ deficiency and PA; the diagnostic and treatment landscape available to clinicians at the time differed substantially from current practice, and participants’ experiences should be interpreted in this historical context. Data were self-reported rather than extracted from medical records, which introduces the possibility of recall bias and prevents independent verification of diagnoses or treatment details from clinics. However, correlations between individual symptoms and reported symptom duration were uniformly weak in magnitude (ranging from −0.032 to 0.205), suggesting limited recall-bias impact on primary findings. The survey lacked formal psychometric validation, and our binary treatment satisfaction measure may not capture nuanced responses. Finally, the cross-sectional design prevents examining symptom evolution or establishing causal relationships, limiting our ability to determine whether symptom subtypes represent stable phenotypes or disease progression stages, or might in part reflect conditions other than B_12_ deficiency.

In conclusion, our identification of three distinct symptom-based subtypes in B_12_ deficiency and PA represents a shift from diagnostic category-driven to symptom pattern-guided approaches. These subtypes, characterized by different symptom burdens, demographic profiles, and treatment response patterns, provide more clinically relevant information than a differential diagnosis of either B_12_ deficiency or PA. The striking finding that standard three-monthly injection protocols showed poor satisfaction across all subtypes (8.5–30.7%), while frequency requirements varied systematically by symptom burden subtype, is consistent with recent NICE guidelines advocating symptom-guided treatment frequency, though the cross-sectional, non-randomized design limits causal inference. For clinicians, these results suggest potential value in systematic symptom assessment and personalized treatment schedules based on symptom subtype, along with heightened vigilance for mental health complications, particularly in severely affected patients. More broadly, this evidence adds to the case for flexible, patient-centered approaches that recognize symptom heterogeneity and by empowering patients as active partners in care decisions; whether such approaches improve outcomes or reduce healthcare utilization remains an important question for future research.

## Data Availability

The data that support the findings of this study were collected by the Pernicious Anaemia Society (PAS) and are not publicly available. The consent provided by participants at the time of data collection did not include permission for public data sharing. Requests for access to the data may be directed to the Pernicious Anaemia Society, brooke_morris@baylor.edu.

## Footnotes

1
https://pernicious-anemia-society.org/

## Glossary

*α*: Alpha; significance threshold
aBIC: Sample-size adjusted Bayesian Information Criterion
AIC: Akaike Information Criterion
AIG: Autoimmune gastritis
B_12_: Vitamin B_12_ (cobalamin)
BIC: Bayesian Information Criterion
CCM: Chronic care model
COVID-19: Coronavirus disease 2019
df: Degrees of freedom
DNA: Deoxyribonucleic acid
EM: Expectation–maximization
FIML: Full information maximum likelihood
HLA: Human leukocyte antigen
IFA: Intrinsic factor antibodies
LCA: Latent class analysis
LMR-LRT: Lo-Mendell-Rubin adjusted likelihood ratio test
MLR: Maximum likelihood ratio
MMA: Methylmalonic acid
NHS: National Health Service
NICE: National Institute for Health and Care Excellence
PA: Pernicious anaemia/anemia
PAS: Pernicious Anaemia Society
PCA: Parietal cell antibodies
QoL: Quality of life
STROBE: Strengthening the Reporting of Observational Studies in Epidemiology
UK: United Kingdom
*χ* ^2^: Chi-square
*ρ*: Spearman rank correlation coefficient
