# Supplementary material for: Patient-reported symptom profiles in vitamin B12 deficiency and pernicious anemia: a cross-sectional study using latent class analysis
Source: Front Med (Lausanne). 2026 Sep 18;13:1927721. doi: 10.3389/fmed.2026.1927721 (PMC13631082; doi:10.3389/fmed.2026.1927721)
Supplement: Supplementary file 1 [file Data_Sheet_1.pdf]

Supplementary Materials

Supplementary Methods……………………………………………………………………………………….…...2

Supplementary Results……………………………………………………………………………………….….4

Supplementary Figure Captions…………………………………………….…………………………………....7

Supplementary Tables……………………………………………….…………………………………...…8

Supplementary Table 1. Pernicious Anaemia Society survey instrument (August 2010-November 2012)…8

Supplementary Table 2. Spearman correlation coefficients between duration of symptoms before diagnosis and individual symptom endorsement……………………………………………………………………10

Supplementary Table 3. Latent Class Analysis model fit statistics for 2-10 class solutions…………….….11

Supplementary Table 4. Age when symptoms first started across symptom burden subtypes…………….…12

Supplementary Table 5. Distribution and satisfaction rates for participants reporting "Other" treatment frequency across symptom burden subtypes…………………………………………….……………….13

Strengthening the Reporting of Observational Studies in Epidemiology (STROBE) Statement…………….…....14

Supplementary References……………………………………………………………………………………....15

Supplementary Methods

Survey Development

The survey instrument was originally developed by the Pernicious Anaemia Society (PAS) in collaboration with a general practitioner (GP) using SurveyMonkey software between August 2010 and November 2012. The survey was designed based on clinical expertise and extensive patient input through the PAS membership, following identification of widespread patient concerns regarding diagnostic delays and treatment dissatisfaction within the patient community.

The comprehensive survey instrument consisted of 38 questions organized into four main sections (**Supplementary Table 1**): 1. Demographics (Part A) is 11 questions covering basic demographic information including sex, age at diagnosis, ethnicity, eye and hair color, family history of pernicious anemia, and dietary patterns (vegetarian/vegan status). 2. Symptom Assessment (Part B) covers systematic documentation of symptoms experienced prior to diagnosis across seven physiological domains: general symptoms (10 items), neurological symptoms (13 items), respiratory symptoms (1 item), cardiovascular symptoms (1 item), gastrointestinal symptoms (6 items), genitourinary symptoms (1 item), and emotional symptoms (4 items). 3. Comorbid conditions (Part C) comprised of 21 questions identifying coexisting medical conditions potentially associated with pernicious anemia across eight clinical areas: general, neurological, dermatologic, cardiovascular, musculoskeletal, endocrine, gastrointestinal, and emotional conditions. 4. Clinical assessment and treatment (Part D) are10 questions assessing diagnostic testing procedures, treatment modalities (type, frequency, and delivery method), treatment satisfaction, and patient experience with healthcare providers

Question formats included binary yes/no responses, single-selection multiple choice, multi-selection options (e.g., "tick all that apply"), and ordinal scales. For treatment satisfaction assessment, participants rated their medical care using a 10-point ordinal scale ranging from "Excellent" to "Unreasonable."

Statistical Software and Technical Specifications

All latent class analyses were conducted using Mplus Version 8·0 with full information maximum likelihood estimation using the robust maximum likelihood ratio (MLR) estimator to handle non-normality in binary indicators (1). R Statistical Software (version 4·0·3; R Core Team 2021) was used for descriptive statistics, post-hoc analyses, and data visualization (2).

Missing Data Pattern Analysis

The percentage of missing values was calculated for all variables included in the survey. All LCA candidate variables demonstrate complete or near-complete data across the analytic sample (N=1,117). Missing data were only observed in post-hoc outcome variables (age when symptoms first started, duration of symptoms before diagnosis, and treatment satisfaction). Participants with missing post-hoc data were retained in the LCA but excluded listwise from the relevant post-hock analysis.

Recall Bias Assessment

To address potential recall bias in retrospective symptom reporting, Spearman rank correlations were calculated between symptom duration before diagnosis and individual symptom endorsement. Symptom duration was measured categorically (1 week to >10 years) and converted to months for analysis. While statistical significance was assessed at α=0·05, correlations were considered practically significant only if $|\rho| \geq 0{\cdot}30$, following established guidelines for clinical research.

Latent Class Analysis Model Specifications

Latent class models were estimated using the expectation-maximization (EM) algorithm with 1,000 random starting values to ensure identification of the global maximum likelihood solution. Models were considered converged when the change in log-likelihood between iterations was less than 1×10^{-6}. To address potential local maxima, we examined the 100 best log-likelihood values from the random starts and verified that the final solution was replicated across multiple starting values.

The selection of indicators for latent class analysis followed established methodological guidelines to ensure model stability and interpretability. From the original survey instrument, systematic selection criteria were applied including a prevalence threshold where variables were included only if endorsed by ≥10·0% of the analytic sample (n≥112), following recommendations by Sinha et al. (2021) to prevent identification of classes based solely on rare indicators (3).

Variables were retained if they represented established manifestations of B_{12} deficiency or PA based on clinical literature review. This approach resulted in 46 indicators being included in the analysis, comprising three demographic indicators, 35 symptoms indicators across multiple physiological domains, and eight comorbid condition indicators.

Model Selection Strategy

Model selection was based on multiple fit indices examined simultaneously, including the Akaike Information Criterion (AIC), Bayesian Information Criterion (BIC), and sample-size adjusted BIC (aBIC), where lower values indicate better fit, with BIC providing stronger penalty for model complexity. Statistical tests included the Lo-Mendell-Rubin likelihood ratio test (LMR-LRT), which tests whether a k-class model fits significantly better than a (k-1)-class model, and entropy, which measures classification quality with values closer to 1·0 indicating clearer class separation.

Models with 2–10 classes were estimated and compared through visual examination of information criteria plots to identify points of diminishing returns in model fit improvement (4). The final model was selected based on the convergence of fit indices, LMR-LRT significance patterns, adequate and stable class sizes, and theoretical interpretability of the resulting subtypes.

Post-Hoc Analyses

Age Distribution Analysis

Age when symptoms first started was analyzed as a categorical variable in nine ranges (0-10, 11-20, 21-30, 31-40, 41-50, 51-60, 61-70, 71-80, 81-90) and evaluated using chi-square analysis to test for associations with symptom burden type. To identify which specific age and subtype combination drove any observed associations, standardized residuals were calculated for each age group and subtype combinations; absolute values above 1·96, corresponding to p<0·05 under the standard normal distribution, were treated as statistically significant and are presented in **Figure 2.** The full distribution of participants across age groups and subtypes is presented in **Supplementary Table 4**. Participants with missing data for age when symptoms first started were excluded listwise from the analysis (n=1,013, 90·7% of the analytic sample).

Symptom Duration Analysis

Duration of symptoms before diagnosis was collected using 11 pre-specified ordered response categories (1 week, 2 weeks, 1 month, 2 months, 3 months, 6 months, 1 year, 2 years, 5 years, 10 years, and more than 10 years). For visualization and analysis of diagnostic delays, duration categories were collapsed into broader clinically relevant groups: less than 6 months, 6 months to 2 years, and more than 2 years (**Figure 3**). The full 11-category distribution is presented in **Supplementary Figure 4**. Chi-square analysis with Cramér's V effect size was used to examine associations between symptom duration and symptom burden subtype for both the collapsed (df=4) and full (df=20) category analyses. Participants with missing data for symptom duration were excluded listwise from both analyses (71 of 1,117 participants; n=1,046, 93·6% of the analytic sample).

Treatment Frequency and Satisfaction Analysis

Treatment frequency and satisfaction rates were examined across symptom burden subtypes among participants with a recorded treatment frequency and satisfaction response (n=927). Frequency categories were collapsed where appropriate: weekly or more (daily, more than once a day, and weekly), monthly, two-monthly, three monthly and other. Participants with missing treatment frequency data (n=155, 13·9% of the analytic sample) or missing satisfaction responses (n=37) were excluded listwise from all treatment analyses. Fisher's exact tests with 10,000 Monte Carlo simulations were used to compare satisfaction rates for each category against the three-monthly injections as the reference category, reflecting the standard NHS treatment schedule. This approach was selected over chi-squared testing as some cells had small expected counts.

Free-text responses in the "Other" category (n=125) were examined separately. Each response was coded by injection interval in weeks and categorized into five groups: loading phase (initial loading doses without an established maintenance frequency), very frequent (every two weeks or less, including daily and alternate day), extended standard (every 3-11 weeks), extended interval (every 12 weeks or more), and no treatment or pre-treatment (participants not yet receiving or unable to receive maintenance injections). Satisfaction rates were calculated within each frequency type and subtype among participants providing a Yes or No satisfaction response.

The "Weekly or more" category in Table 3 and the "Very frequent (≤ 2 weeks)" category in **Supplemental Table 5** are mutually exclusive groups. Table 3 presents participants who selected structured frequency options from the survey response list (Daily, More than once a day, or Weekly), pooled into a single "Weekly or more" category due to small cell sizes. **Supplementary Table 5** presents only participants who selected "Other" and provided a free-text description of their injection frequency; these participants did not select any structured frequency option. The "Very frequent' category in Supplementary Table 5 therefore reflects participants whose self-described frequency was equivalent to weekly or more frequent administration, but who chose to describe it in free text rather than selecting a structured response option. The two groups are non-overlapping.

Statistical Significance and Multiple Comparisons

Statistical significance was set at $\alpha = 0{\cdot}05$ for all analyses. P-values are reported to two significant figures (capped at four decimal places) or as $p<0{\cdot}0001$ for highly significant results. Given the exploratory nature of latent class analysis and the large number of post-hoc comparisons, emphasis was placed on effect sizes and clinical interpretability rather than strict multiple comparison corrections.

Supplementary Results

Missing Data Analysis

Complete data were available for all 46 LCA indicator variables across the full analytic sample (N=1,117). Missing data were only observed in post-hoc outcome variables: age when symptoms frost started (9·3%, n=104), duration of symptoms before diagnosis (6·4%, n=71), and treatment satisfaction (8·1%, n=90). Participants with missing post-hoc data were excluded listwise from the relevant post-hoc analyses (**Supplementary Figure 1**).

Missing data analysis revealed minimal missingness across variables included in the latent class analysis. The majority of key variables showed <1·0% missing data, supporting the robustness of the classification, while variables exceeding 10% missingness were excluded from the LCA indicator set. The optional question regarding children having pernicious anemia showed 97·9% missingness, reflecting the survey design where this question was not applicable to many participants. Chi-square tests confirmed that missing data patterns were not significantly associated with participant demographics ($p>0{\cdot}05$), supporting the assumption that data were missing at random within the context of observed variables (**Supplementary Figure 1**).

Recall Bias Assessment

Spearman rank correlations between symptom duration and individual symptom endorsement were examined to assess potential recall bias in retrospective symptom reporting (**Supplementary Table 2, Supplementary Figure 2**). While 33 of 35 symptoms (94·3%) showed statistically significant correlations with symptom duration ($p<0·05$), the magnitude of these correlations was uniformly weak, ranging from -0·032 to 0·205. No correlations exceeded the threshold for practical significance ($|\rho|\geq 0·30$), suggesting that recall bias had minimal impact on symptom reporting and supporting the validity of retrospective symptom data for latent class analysis. The highest correlations were observed for brittle nails with ridges ($\rho=0·205$), shoulder bumps ($\rho=0·195$), and vertigo ($\rho=0·193$), but even these remained well below thresholds for clinical concern.

Latent Class Analysis Model Selection and Diagnostics

Latent class models with 2-10 classes were systematically evaluated using multiple information criteria (**Supplementary Figure 3, Supplementary Table 3**). The 3-class solution emerged as optimal based on several converging indicators. The 3-class model showed substantial improvement over the 2-class solution across all information criteria, with AIC decreasing from 56733 to 55866, BIC from 57200 to 56568, and aBIC from 56904 to 56123. While the 4-class model achieved a slightly lower BIC (56506), the improvement was minimal and not statistically significant. The LMR-LRT confirmed significant improvement from 2 to 3 classes ($p<0·0001$) but showed no significant improvement from 3 to 4 classes ($p=0·51$), supporting the 3-class solution.

The 3-class model demonstrated high entropy (0·86), indicating clear separation between classes and robust participant classification. The solution produced three distinct and interpretable symptom burden subtypes with adequate sample sizes (High Burden; n=334, Moderate Burden; n=613, Low Burden; n=170. The final 3-class solution demonstrated robust statistical properties, with model convergence achieved across all 1,000 random starting values and consistent replication of the highest log-likelihood value, confirming identification of the global maximum likelihood solution.

Age Distribution Analysis

Significant differences in age when symptoms first started were observed across the three symptom burden subtypes ($\chi^2=37·07$, df=16, $p=0·002$, Cramér's V=0·14; **Figure 2**, **Supplementary Table 4**). The Low Burden subtype showed overrepresentation in older age groups, particularly ages 71-80 (7·7% vs. 3·2% overall) and 61-70 (13·5% vs. 8·4% overall). The High Burden subtype showed overrepresentation in the 31-40 age group (28·5% vs 24·2% overall) and underrepresentation in the 71-80 age group (1·0% vs. 3·2% overall). The Moderate Burden subtype demonstrated the most balanced age distribution, closely matching the overall sample demographics across all age categories.

Symptom Duration and Diagnostic Delay Analysis

Analysis of symptom duration before diagnosis revealed an inverse association between symptom burden and diagnostic timeliness (**Figure 3**, **Supplementary Figure 4**), where participants with the highest symptom burden also experienced the longest pre-diagnosis periods. The High Burden subtype showed particularly prolonged pre-diagnosis periods, with 56·9% of participants experiencing symptoms for more than two years before diagnosis. Conversely, the Low Burden subtype was diagnosed more rapidly, with 21·8% receiving a diagnosis within six months of symptom onset. A clear gradient was observed across subtypes, with diagnostic delays systematically increasing with symptom burden ($\chi^2=115·47$, df=4, $p<0·001$, Cramér's V=0·23, n=1,046). The full 11-category distribution confirmed the pattern ($\chi^2=149·59$, df=20, $p<0·001$, Cramér's V=0·27), though interpretation should be made with caution as some cells had small expected counts.

Treatment Frequency and Satisfaction Analysis

Treatment frequency and satisfaction data were available for 927 of 1,117 participants (82·8%; 155 excluded for missing frequency data, 37 for missing satisfaction response). Three-monthly injections were the most common treatment frequency across all subtypes (High Burden: 42·8%; Moderate Burden: 51·1%; Low Burden: 54·3%),yet showed consistently low satisfaction rates (High Burden: 8·5%; Moderate Burden: 23·7%; Low Burden: 31·1%).

In the High Burden subtype, weekly or more (40·0%, p<0·001), monthly (34·6%, p<0·001), and Other (45·9%, p<0·001) frequencies were all associated with significantly higher satisfaction than three-monthly injections. Two-monthly injections approached but did not reach significance (20·5%, p=0·063). In the Moderate Burden subtype, all frequencies showed significantly higher satisfaction than three-monthly injections (weekly or more: 54·5%, p=0·001; monthly: 48·5%, p<0·001; two-monthly: 47·6%, p<0·001; Other: 58·8%, p<0·001). The Low Burden subtype showed a different pattern, with only monthly (57·9%, p=0·037) and Other (73·3%, p=0·002) frequencies reaching significance compared to three-monthly injections; weekly or more (62·5%, p=0·115) and two-monthly (47·6%, p=0·197) did not differ significantly from three-monthly injections in this subtype.

Analysis of the 125 participants selecting "Other" treatment frequency revealed distinct patterns acroass subtypes (Supplemental Table S5). Very frequent injections (every two weeks or less) were associated with the highest satisfaction rates in the High Burden (77·8%, n=9) and Moderate Burden (81·8%, n=12) subtypes. No High Burden participants reported extended interval injections (every 12 weeks or more). Among the five High Burden with no established treatment, satisfaction was 0·0%, reflecting unmet treatment need in the highest symptom burden group. Loading phase participants shows moderate satisfaction across High Burden (42·9%) and Moderate Burden (42·9%) subtypes, consistence with treatment being in an early transitional stage.

The overlap in apparent frequency between the "Weekly or more" category in Table 3 and the "Very frequent (≤ 2 weeks)" category in Supplementary Table 5 reflects a survey design feature rather than double-counting. Participants in Table 3 selected structured frequency options, while those in Supplementary Table 5 selected "Other" and described equivalent frequencies in free text. Combined, these groups suggest that high-frequency administration, whether formally categorized or self-described, is associated with higher satisfaction rates, particularly in the High Burden subtype.

Supplementary Figure Captions

Supplementary Figure 1. Missing data for post-hoc outcome variables. Percentage of missing values for post-hoc outcome variables in the analytic sample (N=1,117). Complete data were available for all 46 LCA indicator variables (symptoms, conditions, and family history variables) across the full analytic sample. Missing data were only observed in post-hoc outcome variables: age when symptoms first started (9.3%, n=104), duration of symptoms before diagnosis (6.4%, n=71), and treatment satisfaction (8.1%, n=90). Participants with missing post-hoc data were retained in the LCA but excluded listwise from the relevant post-hoc analysis.
Alt text: Horizontal bar chart showing percentage of missing data for three post-hoc outcome variables. Age when symptoms first started has 9.3% missing (n=104), treatment satisfaction has 8.1% missing (n=90), and duration of symptoms before diagnosis has 6.4% missing (n=71). Bars are dark blue. All 46 LCA indicator variables had complete data.

Supplementary Figure 2. Spearman correlation coefficients. Spearman correlation coefficients between symptom duration before diagnosis and individual symptom endorsement for 35 symptoms (N=1,117). Dark blue bars represent statistically significant correlations ($p<0.05$), while light blue bars indicate non-significant correlations. While 33/35 symptoms (94.3%) showed statistically significant correlations, the magnitude was weak (range -0.032 to 0.205), suggesting minimal practical impact of recall bias on symptom reporting.
Alt text: Horizontal bar chart showing Spearman correlation coefficients (rho) between symptom duration before diagnosis and 35 individual symptoms, ordered by correlation magnitude. Dark blue bars indicate statistically significant correlations (p less than 0.05) and light blue bars indicate non-significant correlations. Values range from -0.032 for weight loss to 0.205 for brittle nails with ridges. Thirty-three of 35 symptoms show significant but weak correlations.

Supplementary Figure 3. Model fit statistics for latent class analysis (N=1,117). Information criterion values (AIC, BIC, aBIC) for latent class models with 2-10 classes. Lower values indicate better model fit, with BIC penalizing model complexity more strongly than AIC and aBIC. The 3-class solution (indicated by dashed vertical line) was selected based on convergence of BIC and aBIC inflection points, a significant Lo-Mendell-Rubin test ($p<0.0001$), and adequate class size across all three groups. BIC showed substantial improvement from the 2-class (57200.10) to 3-class model (56568.60), with diminishing returns thereafter. While the 4-class model achieved a lower BIC value (56506.08), the Lo-Mendell-Rubin test indicated no significant improvement over the 3-class solution ($p=0.51$), supporting the 3-class solution as the optimal balance model fit and parsimony. AIC = Akaike Information Criterion; BIC = Bayesian Information Criterion; aBIC = sample-size adjusted BIC.
Alt text: Line graph showing three information criterion values (AIC in medium blue, BIC in dark blue, aBIC in dark red) plotted against number of latent classes from 2 to 10. All three lines show an elbow pattern with the steepest decline between 2 and 3 classes. A vertical dashed dark blue line marks the selected 3-class solution. BIC values range from approximately 57200 at 2 classes to approximately 54838 at 10 classes.

Supplementary Figure 4. Duration of symptoms before diagnosis across symptom burden subtypes ~ full distribution (High Burden n=318, Moderate Burden n=572, Low Burden n=156, total n=1,046)**.** Percentage distribution of symptom duration before diagnosis across the three symptom burden subtypes, showing all 11 duration categories from 1 week to more than10 years. The High Burden subtype (dark red) shows the highest proportion of participants with prolonged symptom duration, particularly in the 5- year and 10+ year categories, while the Low Burden subtype (dark blue) demonstrates earlier diagnosis with higher proportions in shorter duration categories. Chi-square analysis confirmed significant association between symptom duration and class membership ($\chi^2=149.59$, df=20, $p<0.001$, Cramér's V=0.27). Participants with missing data for symptom duration were excluded listwise (71 of 1,117 participants).
Alt text: Grouped bar chart showing percentage of participants in three symptom burden subtypes across three collapsed duration categories: less than 6 months, 6 months to 2 years, and more than 2 years. Bars are dark red for High Burden, medium blue for Moderate Burden, and dark blue for Low Burden. High Burden subtype shows the highest proportion in the more than 2 years category, while Low Burden shows the highest proportion in the less than 6 months category.

Supplementary Tables

Supplementary Table 1. Pernicious Anaemia Society survey instrument (August 2010 – November 2012). The survey comprises four sections: Part A collects demographic information; Part B documents symptoms experienced prior to diagnosis; Part C identifies comorbid conditions potentially associated with pernicious anaemia; and Part D assesses diagnostic testing, treatment modalities, and patient satisfaction with care.

Question No.	Question	Response Options
**Part A: Demographics**		
**1**	Are you?	a. M b. F
**2**	Have you been diagnosed as being deficient in Vitamin B12?	a. Yes b. No
**3**	Have you been formally diagnosed with Pernicious Anaemia?	a. Yes b. No
**4**	How old were you when you had experienced symptoms before being diagnosed?	a. 0–10 d. 31–40 g. 61–70 b. 11–20 e. 41–50 h. 71–80 c. 21–30 f. 51–60 i. 81–90
**5**	For how long had you had symptoms before being diagnosed?	a. 1 week d. 2 months g. 1 year j. 10 years b. 2 weeks e. 3 months h. 2 years k. 10 years+ c. 1 month f. 6 months i. 5 years
**6**	Please specify your ethnic origin:	a. Bangladeshi d. Black – Other g. Pakistani j. Other b. Black – African e. Chinese h. White – European k. Would prefer not to say c. Black – Caribbean f. Indian i. White – Other
**7**	Are you?	a. Vegetarian c. Following a gluten-free diet b. Vegan d. None of these
**8**	Did or do any of the following have Pernicious Anaemia?	a. Mother d. Sister g. Aunt j. Cousin b. Father e. Child h. Grandfather k. None of these c. Brother f. Uncle i. Grandmother
**9**	What colour are your eyes?	a. Blue c. Brown e. Grey b. Green d. Hazel f. Blue-green
**10**	What colour is your hair?	a. Blonde c. Red e. Mousey b. Black d. Brown f. Grey
**11**	If grey, at what age did your hair turn grey?	a. Teens c. 30s e. 50s b. 20s d. 40s f. 60s
**Part B: Please tick any symptoms you experienced prior to diagnosis.**		
**1**	General Symptoms:	a. Brittle nails b. Jaundice c. Dry skin d. Flushed/fever e. Weight loss f. Brittle nails with ridges g. Waking up tired h. Hair loss often with premature greying i. Swollen "beefy" and sore tongue (glossitis) j. Tiredness/continual exhaustion (The 'Strange Tiredness')
**2**	Neurological Symptoms:	a. Forgetfulness/memory loss b. Pins and needles (paraesthesia) c. Poor concentration d. Dizziness e. Headaches f. Vertigo g. Poor sleep (including insomnia) h. Burning legs and feet (Grierson Gopalan Syndrome) i. Inability to stand up with eyes closed (especially when showering) j. Frequently bumping into walls (the Shoulder Bumps) k. Confusion ('handbag in the fridge syndrome') l. Nominal aphasia (forgetting name of objects) m. Clumsiness/lack of coordination
**3**	Respiratory Symptoms:	a. Shortness of breath ('The Sighs')
**4**	Cardiovascular Symptoms:	a. Palpitations
**5**	Gastrointestinal Symptoms:	a. Indigestion b. Stomach cramps c. Loss of appetite d. Loss or changes in taste e. Unexpected and

		unexplained diarrhoea f. Diarrhoea often following a period of constipation
**6**	Genitourinary Symptoms:	a. Bladder/kidney infections
**7**	Emotional Symptoms:	a. Irritability/frustration b. Impatience/desire isolation c. Sudden mood swings d. Suicidal thoughts
**8**	If none of the above apply, please tick none below:	a. None
**Part C: Do you suffer from any of the following medical conditions associated with PA?**		
**1**	General:	a. Tinnitus (ringing/screeching/whistling in the ear) b. Folic acid deficiency
**2**	Neurological:	a. Multiple sclerosis
**3**	Dermatology:	a. Coeliac disease – sensitivity to wheat and/or wheat products b. Vitiligo – white patches that develop on the skin c. Acne rosacea – a reddening of the skin and around the nose d. Psoriasis/eczema/acne e. Psoriatic arthritis
**4**	Cardiovascular:	a. Arrhythmia – irregular, fast or slow heartbeat
**5**	Musculoskeletal:	a. Psoriatic arthritis b. Rheumatoid arthritis
**6**	Endocrine:	a. Diabetes b. Hypothyroidism (underactive) c. Hyperthyroidism (overactive) d. Hyperparathyroidism
**7**	Gastrointestinal:	a. Previous H. pylori infection b. Coeliac disease (gluten/wheat intolerance) c. Stomach ulcers d. Gastrectomy e. Gastric cancer
**8**	Emotional:	a. Depression
**9**	If none of the above apply, please tick none below:	a. None
**Part D: Patient satisfaction with diagnosis and treatment.**		
**1**	What tests did you have when you were diagnosed? If unsure, leave blank:	a. B12 level i. Normal (B12) ii. Low (B12) b. Folic acid level i. Normal (Folate) ii. Low (Folate) c. Intrinsic factor antibodies i. Negative (IFA) ii. Positive (IFA) d. Parietal cell antibodies i. Negative (PCA) ii. Positive (PCA) e. Endoscopy i. Normal (OGD) ii. Abnormal (OGD)
**2**	Please tick what type of B12 replacement therapy you use:	a. Methylcobalamin b. Hydroxocobalamin c. Cyanocobalamin
**3**	How do you receive treatment?	a. Intra-muscular injections b. Sub-cut injections c. Tablets d. Sublingual drops e. Sublingual tablets f. Sublingual spray g. Behind the ear patches h. Nasal spray
**4**	If you have an injection, who injects you?	a. I self-inject b. Private clinic c. Regular health clinic d. Member of the family or family friend
**5**	Frequency of injections:	a. Three monthly b. Two monthly c. Monthly d. Weekly e. Daily f. More than once a day g. Other
**6**	If other, please specify:	Free text response
**7**	Are you satisfied with your current treatment regimen?	a. Yes b. No
**8**	Think about the way in which your condition was investigated and diagnosed. How do you rate your medical care?	a. Excellent b. Very good c. Good d. Adequate e. Reasonable f. Undecided g. Very poor h. Poor i. Inadequate j. Unreasonable
**9**	Were you diagnosed with another disease before being diagnosed as having PA or B12 deficiency?	a. Yes b. No
**10**	If you answered Yes, please state what your doctor thought you were suffering from:	Free text response

Supplementary Table 2. Spearman correlation coefficients between duration of symptoms before diagnosis and individual symptom endorsement (N=1,117). Values represent Spearman correlation coefficients (ρ) and corresponding p-values for 35 symptoms. Duration of symptoms before diagnosis was measured categorically (1 week to >10 years) and converted to months for analysis. Statistically significant correlations ($p<0.05$) were observed for 33 of 35 symptoms; however, all coefficients were weak in magnitude (range -0.032 to 0.205), suggesting minimal practical impact of recall bias on symptom reporting.

**Symptom**	**ρ**	***p-value***
Tiredness	0.076	*0.014*
Hair loss or greying	0.157	*<0.001*
Brittle nails	0.092	*0.003*
Brittle nails with ridges	0.205	*<0.001*
Weight loss	-0.032	*0.302*
Wake up tired	0.125	*<0.001*
Dry skin	0.138	*<0.001*
Flushes fever	0.124	*<0.001*
Glossitis	0.169	*<0.001*
Poor concentration	0.115	*<0.001*
Pins & needles (paresthesia)	0.126	*<0.001*
Confusion	0.156	*<0.001*
Nominal aphasia	0.179	*<0.001*
Clumsiness	0.156	*<0.001*
Headaches	0.163	*<0.001*
Grierson Gopalan Syndrome (burning legs and feet)	0.159	*<0.001*
Forgetfulness/memory loss	0.126	*<0.001*
Poor sleep	0.103	*0.001*
Dizziness	0.160	*<0.001*
Shoulder bumps	0.195	*<0.001*
Can't stand eyes closed	0.144	*<0.001*
Vertigo	0.193	*<0.001*
Shortness of breath (The sighs)	0.113	*<0.001*
Palpitations	0.184	*<0.001*
Indigestion	0.181	*<0.001*
Loss of appetite	0.043	*0.168*
Taste loss or change	0.069	*0.026*
Stomach cramps	0.148	*<0.001*
Diarrhea	0.169	*<0.001*
Diarrhea alternating with constipation	0.154	*<0.001*
Bladder/kidney infections	0.104	*0.001*
Irritability	0.159	*<0.001*
Impatience	0.163	*<0.001*
Sudden mood swings	0.086	*0.005*
Suicidal thoughts	0.176	*<0.001*

Supplementary Table 3. Latent Class Analysis model fit statistics for 2-10 class solutions (N=1,117). BIC, AIC, and aBIC are presented alongside entropy values and Lo-Mendell-Rubin likelihood ratio test (LMR-LRT) p-values for each solution. While AIC and aBIC continue to decrease across all models, BIC shows a clear inflection point at the 3-class solution, supporting selection of this model as the optimal balance between fit and parsimony. Entropy remained high across all solutions (range 0.80-0.87), indicating good classification quality throughout. The LMR-LRT indicated significant improvement adding a third class ($p<0.0001$) but non-significant improvement for the 4-class ($p=0.51$), further supporting a 3-class solution. Class sizes for the selected 3-class solution were adequate (High Burden: n=334, Moderate Burden: n=613, Low Burden: n=170). BIC = Bayesian Information Criterion; AIC = Akaike Information Criterion; aBIC = sample-size adjusted BIC; LMR-LRT = Lo-Mendell-Rubin adjusted Likelihood Ratio Test; ·· = not applicable.

**Model**	**BIC**	**AIC**	**aBIC**	**Entropy**	**LMR**	**Class Size**									
2	57200.10	56733.39	56904.71	0.87	*<0.0001*	520	597	··	··	··	··	··	··	··	··
3	56568.60	55866.02	56123.92	0.86	*<0.0001*	170	334	613	··	··	··	··	··	··	··
4	56506.08	55567.64	55912.12	0.84	*0.51*	125	397	474	121	··	··	··	··	··	··
5	56538.74	55364.43	55795.49	0.80	*0.29*	183	129	343	144	318	··	··	··	··	··
6	56624.53	55214.48	55732.00	0.80	*0.45*	185	139	130	139	302	222	··	··	··	··
7	56756.29	55110.26	55714.48	0.82	*0.40*	149	125	109	302	191	106	135	··	··	··
8	56893.95	55012.05	55702.85	0.82	*0.76*	101	248	123	111	92	142	165	135	··	··
9	57039.38	54921.61	55698.99	0.82	*0.81*	118	100	126	229	98	131	150	137	28	··
10	57191.91	54838.28	55702.24	0.83	*0.80*	111	120	159	28	107	135	97	118	149	93

Supplementary Table 4. Age when symptoms first started across symptom burden subtypes (n=1,013). Values represent number of participants (percentage within subtype) for each 10-year age category. Participants with missing data for age when symptoms first started were excluded listwise (104 of 1,117 participants). Significant differences in age distribution were observed across classes ($\chi2$=37.07, df=16, p=0.002, Cramér's V=0.14). The Low Burden subtype showed significant overrepresentation in older age groups, particularly ages 61-70 (13.5% vs. 8.4% overall) and 71-80 (7.7% vs. 3.2% overall). The High Burden subtype showed overrepresentation in the 31-40 age group (28.5% vs 24.2% overall) and underrepresentation in the 71-80 age group (1.0% vs. 3.2% overall). The Moderate Burden subtype demonstrated the most balanced age distribution across all age categories.

Age Group	High Burden	Moderate Burden	Low Burden
0-10	1 (0.3%)	3 (0.5%)	2 (1.3%)
11-20	7 (2.4%)	15 (2.7%)	1 (0.6%)
21-30	45 (15.3%)	82 (14.6%)	19 (12.3%)
31-40	84 (28.5%)	128 (22.7%)	33 (21.3%)
41-50	91 (30.8%)	160 (28.4%)	36 (23.2%)
51-60	46 (15.6%)	104 (18.5%)	27 (17.4%)
61-70	17 (5.8%)	47 (8.3%)	21 (13.5%)
71-80	3 (1.0%)	17 (3.0%)	12 (7.7%)
81-90	1 (0.3%)	7 (1.2%)	4 (2.6%)

Supplementary Table 5. Distribution and satisfaction rates for participants reporting "Other" treatment frequency across symptom burden subtypes (N=125). Free-text treatment frequency responses from participants selecting "Other" in Table 3 were categorized into five clinically meaningful groups based on injection interval: loading phase, very frequent (every two weeks or less), extended standard (every 3-11 weeks), extended interval (every 12 weeks or more), and no treatment or pre-treatment. Satisfaction rates are presented as number satisfied/number responding (percentage), excluding participants who did not respond to the satisfaction question (High Burden: n=37; Moderate Burden: n=69; Low Burden: n=14 with Yes or No satisfaction response). Very frequent injections (every two weeks or less) were associated with the highest satisfaction rates across all subtypes reporting this frequency (High Burden: 77.8%; Moderate Burden: 81.8%). No High Burden participants reported extended interval injections (every 12 weeks or more), while no High Burden participants with no established treatment reported satisfaction (0.0%, n=5). The single Moderate Burden participant with no treatment reporting satisfaction (100.0%) should be interpreted with caution given the small cell size. Loading phase satisfaction reflects treatment at an early stage where a maintenance frequency has not yet been established.

	High Burden (n=39)		Moderate Burden (n=72)		Low Burden (n=14)	
**Treatment Frequency**	**n**	**n satisfied (%)**	**n**	**n satisfied (%)**	**n**	**n satisfied (%)**
Loading phase	9	3/7 (42.9%)	14	6/14 (42.9%)	NA	NA
Very frequent (≤2 weeks)	9	7/9 (77.8%)	12	9/11 (81.8%)	1	1/1 (100.0%)
Extended standard (3-11 weeks)	16	7/16 (43.8%)	35	19/34 (55.9%)	8	6/8 (75.0%)
Extended interval (≥12 weeks)	NA	NA	9	5/9 (55.6%)	5	4/5 (80.0%)
No treatment or pre-treatment	5	0/5 (0.0%)	2	1/1 (100.0%)	NA	NA

Additional file 12. STROBE Statement ~ Checklist of items that should be included in reports of cross-sectional studies.

Item	Item No	STROBE Guideline
**Title and Abstract**		
	1	(a) Indicate the study's design with a commonly used term in the title or the abstract. (b) Provide in the abstract an informative and balanced summary of what was done and what was found.
**Introduction**		
Background/rationale	2	Explain the scientific background and rationale for the investigation being reported.
Objectives	3	State specific objectives, including any prespecified hypotheses.
**Methods**		
Study design	4	Present key elements of study design early in the paper.
Setting	5	Describe the setting, locations, and relevant dates, including periods of recruitment, exposure, follow-up, and data collection.
Participants	6	Cross-sectional study - Give the eligibility criteria, and the sources and methods of selection of participants.
Variables	7	Clearly define all outcomes, exposures, predictors, potential confounders, and effect modifiers. Give diagnostic criteria, if applicable.
Data sources/measurements	8	For each variable of interest, give sources of data and details of methods of assessment (measurement). Describe comparability of assessment methods if there is more than one group.
Bias	9	Describe any efforts to address potential sources of bias.
Study size	10	Explain how the study size was arrived at.
Quantitative variables	11	Explain how quantitative variables were handled in the analysis. If applicable, describe which groupings were chosen and why.
Statistical methods	12	(a) Describe all statistical methods, including those used to control for confounding.<br>(b) Describe any methods used to examine subgroups and interactions.<br>(c) Explain how missing data were addressed.<br>(d) Cross-sectional study - If applicable, describe analytical methods taking account of sampling strategy.<br>(e) Describe any sensitivity analysis.
**Results**		
Participants	13	(a) Report numbers of individuals at each stage - e.g., numbers potentially eligible, examined for eligibility, confirmed eligibility, included in the study, completing follow-up, and analyzed.<br>(b) Give reasons for non-participation at each stage.<br>(c) Consider use of a flow diagram.
Descriptive data	14	(a) Give characteristics of study participants (e.g., demographic, clinical, social) and information on exposures and potential confounders.<br>(b) Indicate number of participants with missing data for each variable of interest.
Outcome data	15	Cross-sectional study - Report numbers of outcome events or summary measures.
Main results	16	(a) Give unadjusted estimates and, if applicable, confounder-adjusted estimates and their precision (e.g., 95% confidence interval). Make clear which confounders were adjusted for and why they were included.<br>(b) Report category boundaries when continuous variables were categorized.<br>(c) If relevant, consider translating estimates of relative risk into absolute risk for a meaningful time period.
Other analysis	17	Report other analyses done - e.g., analysis of subgroups and interactions and sensitivity analyses.
**Discussion**		
Key results	18	Summarize key results with reference to study objectives.
Limitations	19	Discuss limitations of the study, taking into account sources of potential bias or imprecision. Discuss both direction and magnitude of any potential bias.

Interpretation	20	Give a cautious overall interpretation of results considering objectives, limitations, multiplicity of analyses, results from similar studies, and other relevant evidence.
Generalizability	21	Discuss the generalizability (external validity) of study results.
**Other information**		
Funding	22	Give the source of funding and the role of the funders for the present study and, if applicable, for the original study on which the present article is based.

Supplementary References

1. Muthén LK, Muthén BO. Mplus_Users_Guide.pdf.

2. Team RC. *R: A language and environment for statistical computing*. Vienna, Austria: R Foundation for Statistical Computing. (2021). https://www.R-project.org/

3. Sinha P, Calfee CS, Delucchi KL. Practitioner's Guide to Latent Class Analysis: Methodological Considerations and Common Pitfalls. *Crit Care Med* (2020) 49:e63–e79. doi: 10.1097/ccm.0000000000004710

4. Nylund KL, Asparouhov T, Muthén BO. Deciding on the Number of Classes in Latent Class Analysis and Growth Mixture Modeling: A Monte Carlo Simulation Study. *Struct Equ Model: A Multidiscip J* (2007) 14:535–569. doi: 10.1080/10705510701575396
